# Unconventional Spin–Orbit Torques by 2D Multilayered MXenes for Future Nonvolatile Magnetic Memories

**DOI:** 10.1002/smll.202500626

**Published:** 2025-05-15

**Authors:** Prabhat Kumar, Yoshio Miura, Yoshinori Kotani, Akiho Sumiyoshiya, Tetsuya Nakamura, Gaurav K. Shukla, Shinji Isogami

**Affiliations:** ^1^ Research Center for Magnetic and Spintronic Materials National Institute for Materials Science (NIMS) Sengen 1‐2‐1, Tsukuba Ibaraki 305‐0047 Japan; ^2^ Faculty of Electrical Engineering and Electronics Kyoto Institute of Technology Hashikami‐cho, Matsugasaki, Sakyo‐ku Kyoto 606‐8585 Japan; ^3^ Photon Science Innovation Center (PhoSIC) Aoba 468‐1, Aramaki‐Aza, Aoba Sendai 980‐0845 Japan; ^4^ International Center for Synchrotron Radiation Innovation Smart (SRIS) Tohoku University. Aoba 468‐1, Aramaki‐Aza, Aoba Sendai 980‐8572 Japan

**Keywords:** field‐free switching, MRAM, MXene, unconventional SOT, XMCD

## Abstract

MXenes have attracted attention in recent years owing to their 2D layered structures with various functionalities. To open a new application field for MXenes in the realm of electronic devices, such as ultrahigh‐integrated magnetic memory, a spin–orbit torque (SOT) bilayer structure with MXene of Cr_2_N is developed: substrate//Cr_2_N/[Co/Pt]_3_/MgO using the magnetron sputtering technique. Field‐free current‐induced magnetization switching in the bilayer structure is demonstrated, regardless of the charge current directions with respect to the mirror symmetry lines of Cr_2_N crystal. This is a specific characteristic for the 2D MXene‐based SOT‐devices. As the SOT efficiency increases with increasing the Cr_2_N thickness, the first‐principles calculations predict an intrinsic orbital‐Hall conductivity with the dominant out‐of‐plane component, comparing to the spin‐Hall conductivity in the Cr_2_N. X‐ray magnetic circular dichroism reveals the out‐of‐plane uncompensated magnetic moment of Cr ($m_{\mathrm{Cr}}^{\mathrm{UC}.}$) in the Cr_2_N layer at the interface, induced by contact with the Co in the [Co/Pt]_3_ ferromagnetic layer. Therefore, the intrinsic bulk orbital‐Hall effect in MXene and the interfacial contribution such as spin‐filtering‐like effect owing to $m_{\mathrm{Cr}}^{\mathrm{UC}.}$ are considered as possible major mechanisms for the unconventional out‐of‐plane SOT in the device, rather than a crystal symmetry and/or an interlayer exchange coupling.

## Graphical abstract

MXene is a recent 2D material with practical functionalities. In this study, Cr_2_N, a bare MXene is employed in the nonvolatile memory device as a source of spin–orbit torques. Field‐free current‐induced robust magnetization switching is demonstrated, suggesting the specific characteristics for the MXene that is not realized by the other 2D materials.

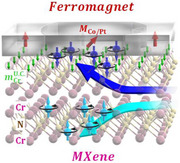

## 1 Introduction

The importance of semiconductor devices is rapidly increasing owing to the development of a modern society, in which humans are connected to all types of applications via the Internet. To realize efficient devices, 2D materials, such as the single‐layer graphene and the transition metal dichalcogenides (TMDC), have attracted significant attention owing to their various functionalities.^[^ ^1^, ^2^, ^3^, ^4^, ^5^, ^6^, ^7^, ^8^, ^9^ ^]^ Furthermore, transition metal carbide Ti_3_C_2_ with atomic layered structures was first discovered in 2011,^[^ ^10^ ^]^ and has opened a new class of 2D materials. It is termed as MXene and is also known as post‐graphene and TMDC.^[^ ^11^ ^]^ The chemical formula is *M* *_n_*_+1_ *X* *_n_* *T* *_x_*, where the site *M* represents the transition metals, such as Ti and Cr, *X* represents the 2*p* light elements C or N, and *T* represents surface terminations such as O and Cl on the outer *M* layer. Specifically, *n* varying from 1 to 4 corresponds to the number of the simplest *M*─*X*─*M* bonding trilayer units of *M*_2_ *X*, while *x* is a variable. The physical and chemical properties can be tailored by *n*, as well as by various combinations of *M*, *X*, and *T*.^[^ ^12^ ^]^ These characteristics are related to the significant electronegativity of 2*p* light element *X*, which allows for the strong orbital hybridization with the *M* elements.^[^ ^13^ ^]^ Thus, MXenes are widely considered to have immense potential as key materials for many device applications. In the past decade, MXenes have contributed to the fields such as biomedicine,^[^ ^14^ ^]^ mechanical science,^[^ ^15^ ^]^ optoelectronics,^[^ ^16^ ^]^ and energy storage.^[^ ^17^ ^]^ These pioneering works inspired our interest in finding more remarkable potential for MXenes. In this study, we aim to develop another application field of MXene by expanding it to the field of 2D spintronics,^[^ ^18^, ^19^ ^]^ which has been unfamiliar with MXene, and elucidated its superiority. Furthermore, we examined the MXene‐specific spin‐transport phenomena beyond charge transport.

In the spintronics research, manipulation of the magnetic moment via the spin degree of freedom has attracted considerable attention in terms of electronic devices with low power consumption because the spin current, a flow of spin angular momentum without electron charge, does not consume power in principle.^[^ ^20^ ^]^ Specifically, to store the enormous amount of data associated with the widespread use of the Internet and mobile applications, nonvolatile spin‐orbit torque magnetic random access memory (SOT‐MRAM) has been extensively studied as one of the storage devices that takes advantage of the spin current.^[^ ^21^ ^]^ Although three types of SOT‐MRAMs with the magnetization directions of the recording layer in *x*, *y* and *z* have been proposed,^[^ ^22^ ^]^ the so‐called Type‐Z with perpendicularly oriented magnetization (in *z* direction) has been identified as a promising geometry for further high‐integration and low‐power MRAM packages. However, the Type‐Z requires an in‐plane bias magnetic field for writing using conventional in‐plane current‐induced SOT, originating from the spin current in conventional heavy metals, such as Ta, Pt, and W. This has been an issue for achieving the miniaturization of cell size and highly integrated SOT‐MRAMs, leading to a recent challenge for field‐free current‐induced magnetization switching (CIMS) by SOT, and many approaches have been demonstrated to date:^[^ ^23^ ^]^ the compositional and/or geometrical gradient in the multilayers;^[^ ^24^, ^25^ ^]^ the interfacial engineering by nonmagnetic layers;^[^ ^26^, ^27^ ^]^ the exchange coupling between antiferromagnet/ferromagnet bilayers;^[^ ^28^, ^29^ ^]^ the crystal symmetricity of noncollinear antiferromagnet and the low‐symmetry TMDC;^[^ ^30^, ^31^, ^32^, ^33^, ^34^, ^35^, ^36^, ^37^ ^]^ and the complex circuit architecture with combined SOT and spin‐transfer‐torque.^[^ ^38^, ^39^ ^]^ These are based on the concepts of superimposing the out‐of‐plane SOT component on a conventional in‐plane SOT, which is referred to as an unconventional SOT.

In addition to the aforementioned artificial structures and materials, we focus on the 2D bare MXene (*M*_2_ *X*) as a spin source layer to realize field‐free CIMS. Although there are many candidates for the *M* sites, such as Ti, V, W, Mo, and Cr, we employed Cr_2_N in terms of the phase stability and metallic conductivity, as in the case of conventional transition metal nitrides.^[^ ^40^, ^41^ ^]^ There are three aspects to be focused on for the MXene‐based SOT‐device. i) A low‐symmetry driven unconventional out‐of‐plane SOT, similar to the noncollinear antiferromagnets and TMDCs,^[^ ^30^, ^31^, ^32^, ^33^, ^34^, ^35^, ^36^, ^37^ ^]^ in which field‐free CIMS can be induced by an in‐plane charge current orthogonal to a mirror symmetry line of the crystal, but not in parallel. ii) An orbital Hall effect (OHE) in the bulk part of MXene layer. The bilayer system with oxidized light elements exhibits an unconventional SOTs in spite of their weak spin‐orbit interaction (SOI),^[^ ^42^, ^43^, ^44^ ^]^ which is discussed based on the transfer of orbital angular momentum. (iii) An interfacial contribution to the out‐of‐plane SOT. Owing to the advantageous properties of 2D materials, the MXene exhibits an atomically flat interface with the ferromagnetic layer, enabling the discernment of interfacial effects such as electronic band structure and interlayer magnetic coupling. Recently, van der Waals 2D heterostructures enable field‐free CIMS by the unconventional SOT,^[^ ^45^ ^]^ which is discussed with the interfacial states such as efficient spin transparency and interfacial magneto‐spin Hall effect.^[^ ^46^ ^]^ Therefore, we have measured the CIMS with two different directions of in‐plane charge current and various thickness of MXene, and we have quantified polarized spins at an interface via synchrotron radiation in this study.

**Figure 1:**
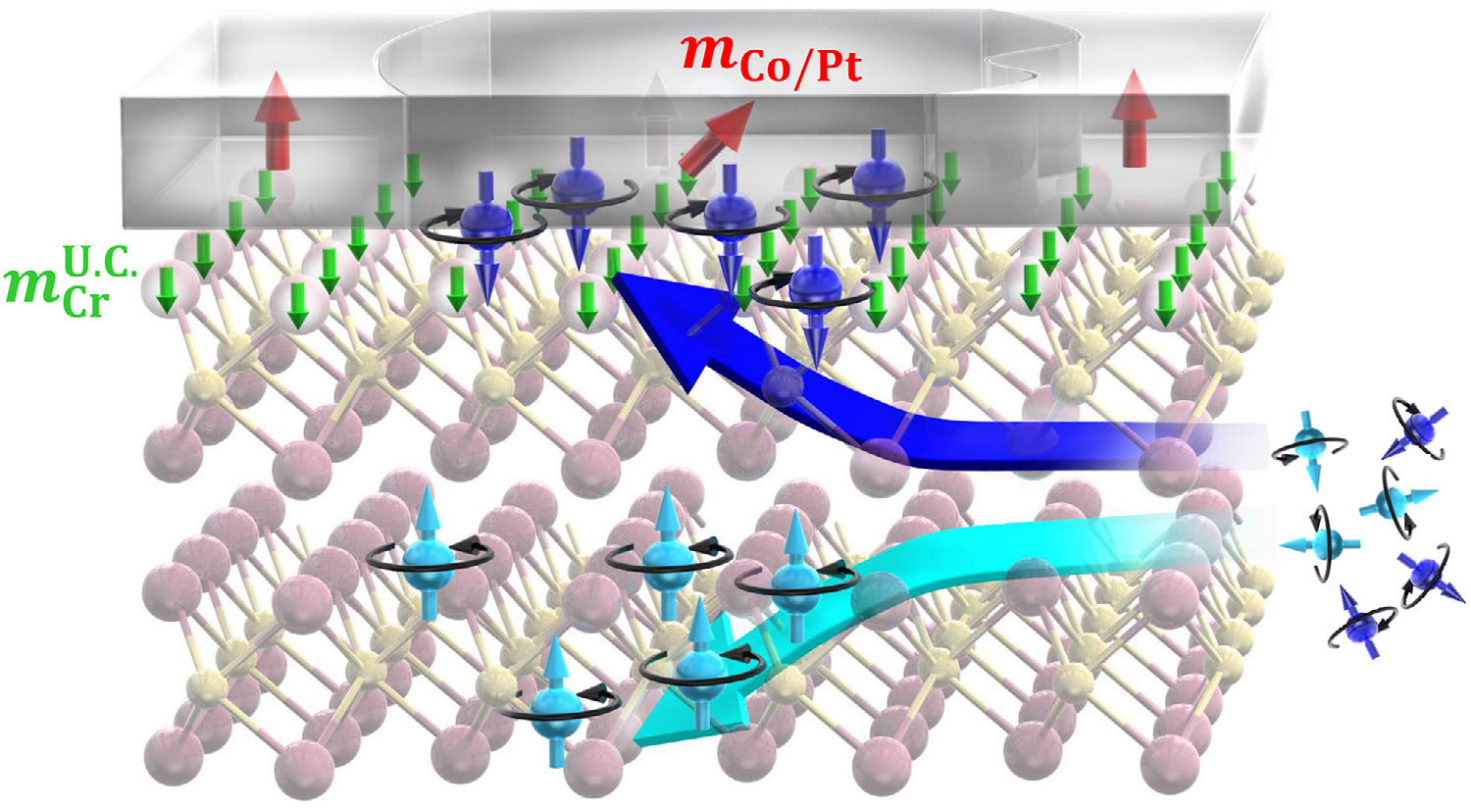
Concept of the field‐free CIMS in the 2D MXene‐based SOT‐device, i.e., the MXene (Cr_2_N)/ferromagnet bilayer system. The electron‐spins oriented in the out‐of‐plane direction emerge through the pronounced orbital Hall effect in the MXene layer. The thick green arrows at the interface represent the out‐of‐plane uncompensated magnetic moment of Cr ($m_{\mathrm{Cr}}^{\mathrm{UC}.}$) induced by the adjacent ferromagnetic layer. The $m_{\mathrm{Cr}}^{\mathrm{UC}.}$ can act as a spin‐filter that transfers the polarized electron‐spins with the same orientation as the $m_{\mathrm{Cr}}^{\mathrm{UC}.}$. The out‐of‐plane oriented spins exert the torque on the ferromagnetic layer, resulting in the magnetic domain switching without in‐plane magnetic fields.

In **Figure** **1**, we depict the possible interpretations of the field‐free CIMS in MXene‐based SOT‐device based on the findings of this study. Firstly, the spin current in the Cr_2_N layer is expected to originate from the OHE with out‐of‐plane component, which dominates the entire SOT exerted on the ferromagnetic layer. Second, the nonmagnetic Cr layer, i.e., the top trilayer‐unit of the Cr_2_N MXene adjacent to the ferromagnetic layer, can be polarized, resulting in an uncompensated magnetic moment of Cr ($m_{\mathrm{Cr}}^{\mathrm{UC}.}$) in the out‐of‐plane direction, as depicted by the thick arrows at the interface. This is primarily responsible for the superposition of the out‐of‐plane SOT component owing to a spin‐filtering‐like mechanism at the interface.^[^ ^27^, ^47^ ^]^ In another words, the spin converted from the orbital angular momentum in the same direction as the $m_{\mathrm{Cr}}^{\mathrm{UC}.}$, can be transferred, while the spin in the opposite direction cannot be transferred. This is a unique mechanism for the field‐free CIMS in the MXene‐based SOT‐devices, which cannot be explained by the existing scenario of 2D crystal symmetry.

## 2 Results and Discussion

**Figure 2:**
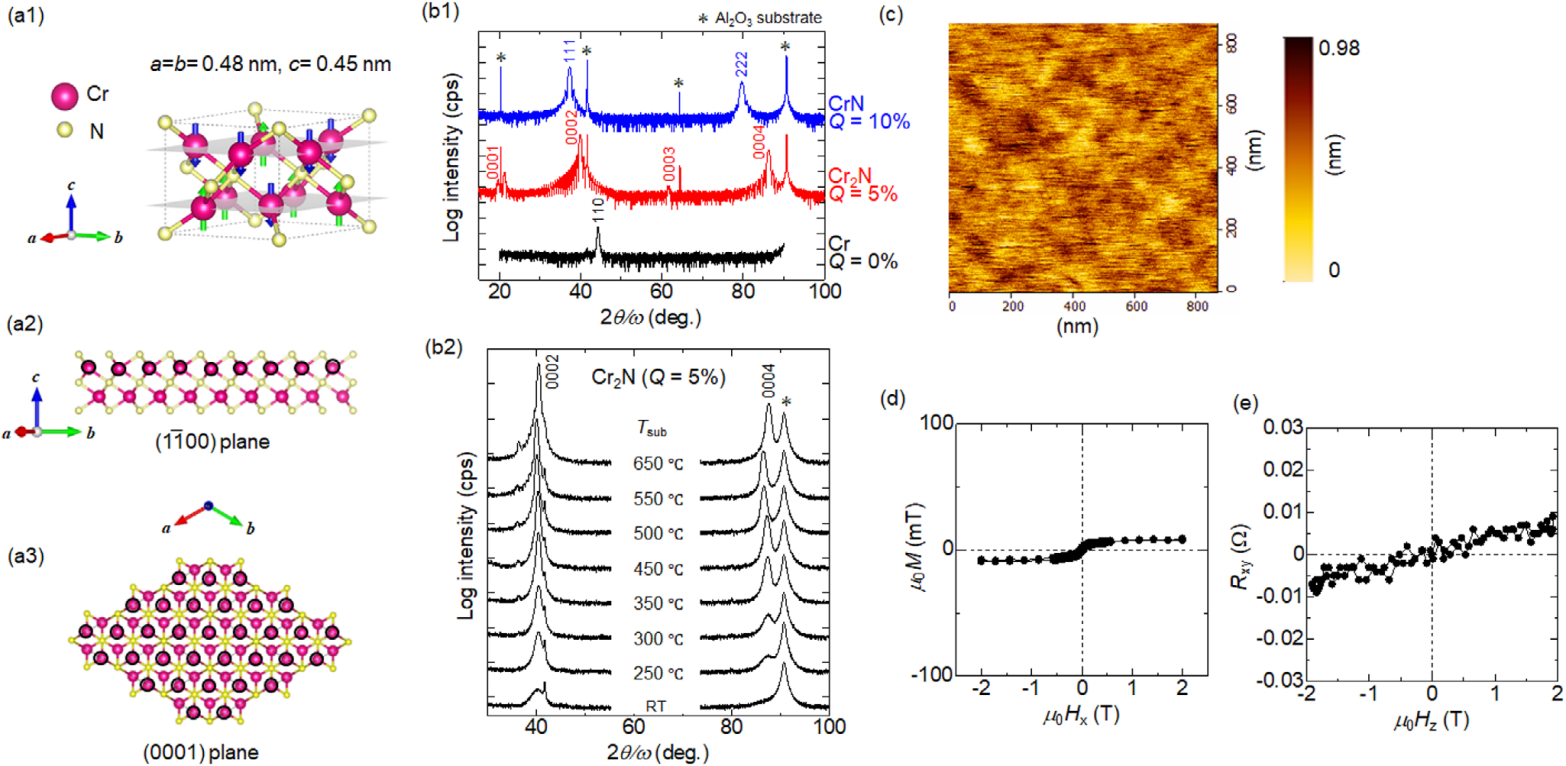
a1) Unit cell model of the Cr_2_N bare MXene together with the possible magnetic structure predicted by first‐principles calculation. a2) Cross‐sectional view and a3) plane view for the 3 × 3 × 1 supercell, where the Cr atoms at the top layer are surrounded by black circles. b1,b2) Out‐of‐plane XRD profiles for the 20 nm thick Cr–N films deposited with different N_2_ flow ratio *Q* = N_2_/(Ar + N_2_) while reactive sputtering deposition, and substrate temperature (*T*_sub_). c) Atomic force microscopy image for the 20‐nm‐thick Cr_2_N film. d) Magnetization curve measured by the in‐plane magnetic field (*H_x_*). e) Anomalous Hall resistance as a function of out‐of‐plane magnetic field (*H_z_*).

The unit cell of Cr_2_N MXene shows a hexagonal structure with a space group of $P\bar{3}1m$, as shown in **Figure** **2**a1, and the lattice constants are *a* = *b* = 0.48 nm and *c* = 0.45 nm. The collinear antiferromagnetic structure has been reported for a wide temperature range from 100 K to 500 K,^[^ ^48^, ^49^, ^50^, ^51^, ^52^ ^]^ which was also predicted by our first‐principles calculations, as indicated by the arrows on each Cr atom. The cross‐sectional and plane views are shown together with their coordinates in Figure 2a2,a3, where the Cr atoms in the top layer are surrounded by the black circles to distinguish them from the bottom Cr layer. The top and bottom Cr layers exhibited a close‐packed structure, and N atoms were present at the octahedral sites between the two Cr layers. Note that the present Cr_2_N belongs to the family of bare MXene without *T* sites, but the Cr‐N‐Cr trilayer unit is bonded via stacking of N layers. Figure 2b1 shows the out‐of‐plane X‐ray diffraction (XRD) profiles of pure Cr, CrN, and Cr_2_N films with a thickness of ∼20 nm on the *c*‐plane oriented Al_2_O_3_ substrate. Each phase can be formed using different ratios of N_2_ gas flow, defined as *Q* = N_2_/(Ar + N_2_), in reactive sputtering deposition. At *Q* = 0%, a pure Cr film was grown with (110) texture. For *Q* = 5%, the fringe oscillation was observed near the XRD peak at 2*θ*/*ω* ≈ 40° and 88°, suggesting the long‐range and stoichiometric Cr_2_N‐MXene phase formation with hexagonal crystal structure with long‐range atomically flat interfaces. For *Q* = 10%, the CrN, which is another phase of the Cr‐N intermetallic compound with face‐centered‐cubic structure, was grown in (111) texture. Figure 2b2 shows the substrate temperature (*T*_sub_) dependence of the out‐of‐plane XRD profiles of Cr_2_N films with the same thicknesses. Although the texture with (0001) orientation can be grown even at room temperature (RT), high atomic order was obtained at *T*_sub_ ranging from 350 °C to 650 °C. Figure 2c shows the atomic force microscope image for the 20‐nm‐thick Cr_2_N film to show the long range flatness. The root mean square roughness was evaluated to be only 0.12 nm, suggesting the atomically flat surface as indicated by the fringe oscillation in Figure 2b1. Figure 2d,e shows the in‐plane magnetic properties (in *x*‐direction) and the anomalous Hall measurements with the magnetic field along the out‐of‐plane direction (in *z*‐direction) of Cr_2_N layer. The magnetization was negligible without hysteresis, which can be attributed to the antiferromagnetism of Cr_2_N, as predicted by first‐principles calculations.^[^ ^48^, ^49^, ^50^, ^51^, ^52^ ^]^

**Figure 3:**
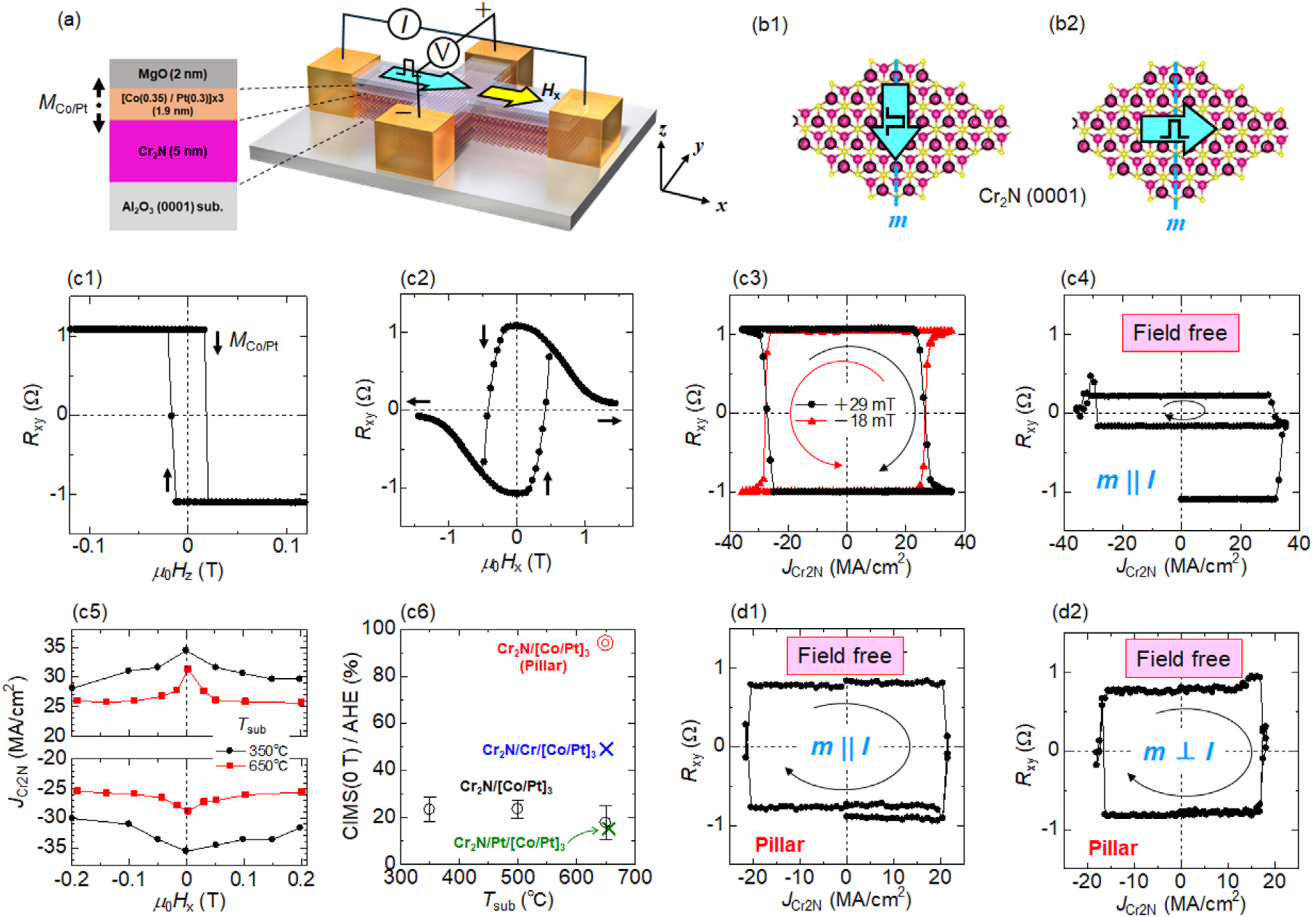
a) Measurement configuration of the current‐induced magnetization switching (CIMS) and representative stacking structure, where the 5 nm thick MXene layer consists of ∼10 unit‐layers of Cr_2_N. b1,b2) Two different directions of the current pulse with respect to mirror symmetry line (*m*) in the CIMS demonstration. c1,c2) Anomalous Hall resistance for the same sample with magnetic field along out‐of‐plane (*H_z_*) and in‐plane directions. c3,c4) Representative CIMS with and without *H_x_*. c5) *H_x_* dependence of *J*_Cr2N_ for the Cr_2_N growth temperature (*T*_sub_) of 350 °C and 650 °C. c6) Field‐free CIMS ratio relative to the full switching by *H*_z_ for various *T*_sub_. The green and blue symbols represent the same results, but with the insertion of 1 nm thick Pt and Cr layers, respectively, for comparison. The red symbol indicates the result with circular shaped pillar devices. d1,d2) Representative field‐free CIMS loops for the pillar device with the current pulses parallel and orthogonal to *m* (see Figure S5 in the Supporting Information).

To investigate the CIMS characteristics owing to Cr_2_N MXene, we prepared SOT‐devices, as depicted in **Figure** **3**a: substrate//Cr_2_N(5 nm)/[Co(0.35 nm)/Pt(0.3 nm)]_3_/MgO(2 nm). The Co/Pt multilayer with three periods, which is described as [Co/Pt]_3_, provides sufficient perpendicular magnetic anisotropy, resulting in an out‐of‐plane magnetization (*M*_Co/Pt_) at the remanent state (see Figure S1 in the Supporting Information). Figure 3b1,b2 exhibit (0001) plane of the Cr_2_N supercell, which is the same as that shown in Figure 2a3, and mirror symmetry axes are depicted by the blue dashed lines (*m*). In order to explore the specific characteristics of Cr_2_N/ferromagnet system that cannot be explained by such mirror symmetry, we intentionally applied a charge current pulse parallel and orthogonal to the mirror axis, in which the out‐of‐plane SOT vanished for only the configuration of parallel (Figure 3b1) in the case of WTe_2_/ferromagnet systems.^[^ ^33^, ^34^ ^]^ The anomalous Hall resistance (*R_xy_*) with the magnetic field sweep along the out‐of‐plane (*H_z_*) and in‐plane (*H_x_*) direction is shown in Figure 3c1,c2, respectively, where *M*_Co/Pt_ is indicated by arrows. The *R_xy_* amplitude for *μ*_0_ *H_z_* = 0 was consistent with that for *μ*_0_ *H_x_* = 0, suggesting sufficient perpendicular magnetic anisotropy in the [Co/Pt]_3_ layer such that *M*_Co/Pt_ points in *z*‐direction at the magnetic remanent state. Figure 3c3 shows the CIMS behaviors under the bias field of *H_x_*, where the load current pulse duration is 10 ms and the direction is parallel to *m* (*m* || *I*) as described in Figure 3b1. The change in *R_xy_*, corresponding to the magnetization switching, was remarkably sharp as observed in the conventional heavy‐metal based SOT‐device with Type‐Y geometry,^[^ ^53^ ^]^ and the polarity of CIMS loop depending on the *H_x_* was clockwise (CW) for *μ*_0_ *H_x_* = +29 mT and counter‐clockwise (CCW) for *μ*_0_ *H_x_* = ‐18 mT. It should be noted that the partial CIMS occurred at *μ*_0_ *H_x_* = 0 (field free) as shown in Figure 3c4, and the effective critical current density flowing in the Cr_2_N layer (*J*_Cr2N_) was ∼30 MA/cm^2^. Specifically, the value was obtained by eliminating the current shunting to the [Co/Pt]_3_ layer based on the measured resistivity of Cr_2_N and [Co/Pt]_3_ layers (see Figure S2 in the Supporting Information). Note that the *J*_Cr2N_ obtained is comparable to that of Type‐Y devices,^[^ ^53^ ^]^ which include heavy‐metals with strong SOI and high SOT efficiency. The polarity of the field‐free CIMS was CW, which was confirmed for the other 10–20 devices. The *J*_Cr2N_‐*H_x_* diagram shown in Figure 3c5 revealed that the *J*_Cr2N_ decreased with increasing *H_x_*, and the field‐free CIMS was achieved even at lower *T*_sub_ = 350 °C (see Figure S3 in the Supporting Information). In the production line of the CMOS transistor, in which the SOT‐MRAMs are to be embedded, post‐annealing has been performed at ∼400 °C. Note that the Cr_2_N based SOT‐devices have enough heat endurance to exhibit a stable CIMS owing to the robustness of the Cr_2_N crystal structure with respect to growth temperature (Figure 2b2). The ratio of field‐free CIMS to the full CIMS was approximately 20%, regardless of *T*_sub_, as shown in Figure 3c6. These intermediate states were also observed in the other devices as well we measured (see Figure S4 in the Supporting Information). Nevertheless, the ratio can be enhanced by the 1‐nm‐thick Cr insertion (blue symbol), while it is slightly suppressed by the 1‐nm‐thick Pt insertion (green symbol), suggesting a relationship between the interfacial phenomena and out‐of‐plane SOT component. Given that [Co/Pt]_3_ ferromagnetic layer of the Hall‐cross device has a large area with 10 µm × 7 µm, multiple domain structures would form when CIMS occurs, resulting in an intermediate magnetic state.^[^ ^54^ ^]^ On the other hand, Figure 3d1 shows the field‐free CIMS loops for the same SOT‐devices with [Co/Pt]_3_ ferromagnetic pillar with ∼7 µm in diameter. Note that the in‐plane charge current flowed along the mirror symmetry line, in which out‐of‐plane SOT is not allowed in principle. The switching ratio was much larger than that with Hall‐cross geometry (Figure 3c4), leading to ∼94% of full switching amplitude (see Figure S5 in the Supporting Information). Figure 3d2 shows the CIMS with the in‐plane charge current orthogonal to the mirror line, in which the out‐of‐plane SOT is allowed in principle. The field‐free CIMS was also observed with similar switching ratio, suggesting an unconventional out‐of‐plane SOT that cannot be explained by the existing symmetry driven out‐of‐plane SOT mechanism.

**Figure 4:**
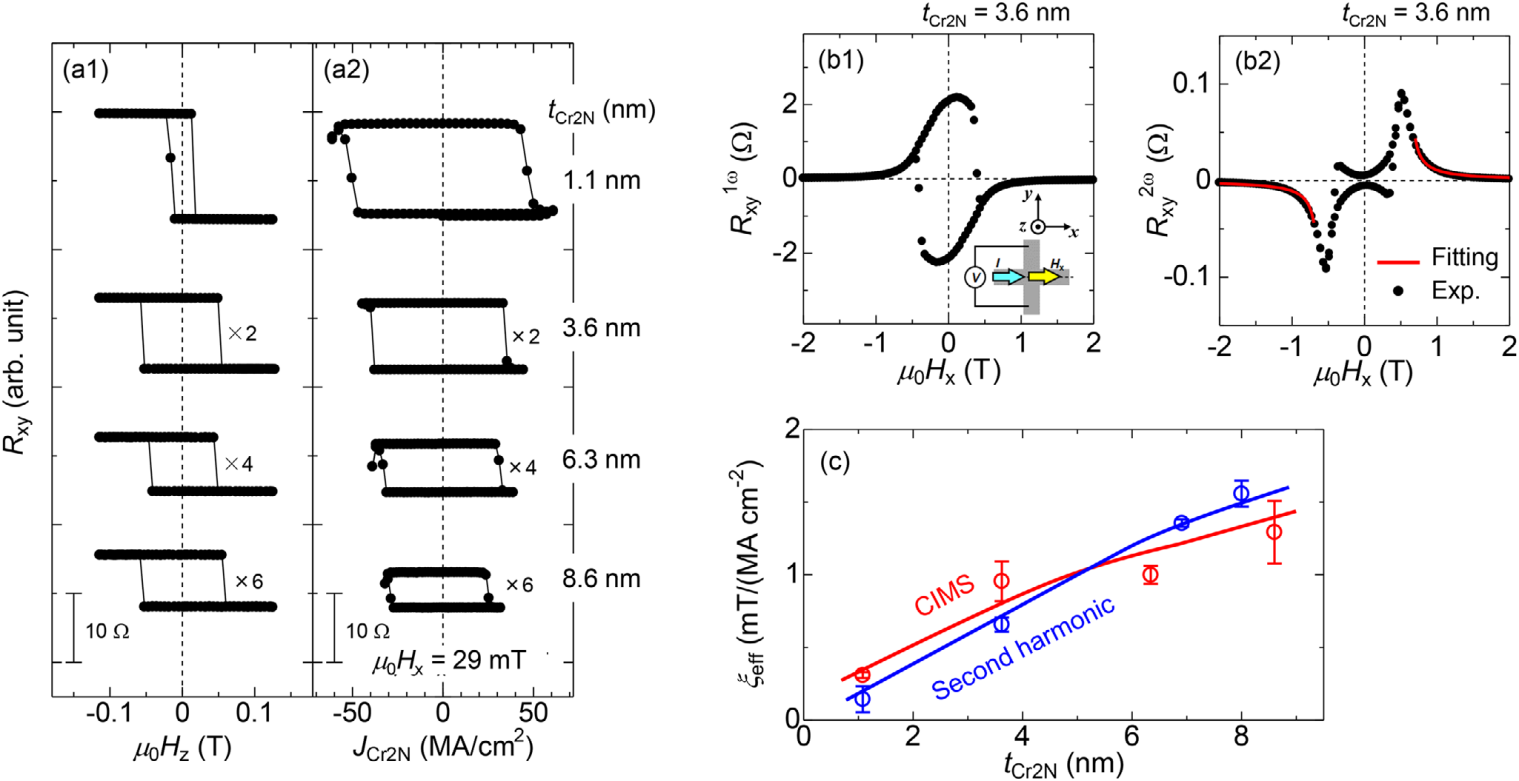
a1,a2) Cr_2_N layer thickness (*t*_Cr2N_) dependence of the out‐of‐plane AHE and CIMS loops with the bias field *μ*_0_ *H_x_* = +29 mT, for the SOT‐device of substrate//Cr_2_N(*t*_Cr2N_)/[Co(0.35 nm)/Pt(0.3 nm)]_3_/MgO(2 nm). b1,b2) High‐field in‐plane AHE loops for the first‐ (1ω) and second‐harmonic (2ω) Hall resistance. The red curve in (b2) represents the fitting result using Equation (2) to distinguish the damping‐like (*H*_DL_) and field‐like (*H*_FL_) SOT effective fields from the magneto thermoelectric effect. c) Damping‐like SOT efficiency (*ξ*_eff_) as a function of *t*_Cr2N_ estimated by both the critical *J*_Cr2N_ in CIMS loops (red) and the *H*_DL_ value in the second‐harmonic measurements (blue).

To assess the possible mechanism of the unconventional SOT, the effective efficiency (*ξ*_eff_) was quantified for the Hall‐cross devices with various Cr_2_N thicknesses: substrate//Cr_2_N(*t*_Cr2N_)/[Co(0.35 nm)/Pt(0.3 nm)]_3_/MgO(2 nm). **Figure** **4**a1,a2 show the out‐of‐plane AHE and CIMS loops, respectively, where the charge current direction was parallel to the mirror symmetry axis (*m* || *I*). The amplitude of *R_xy_* decreased with increasing current‐shunting into the Cr_2_N layer; thus, we multiplied the factors to expand the loops for visibility. The amplitudes of AHE and CIMS were comparable for each *t*_Cr2N_, and the critical current density of *J*_Cr2N_ decreased with increasing *t*_Cr2N_. The *ξ*_eff_ was estimated using the following equation,^[^ ^55^, ^56^ ^]^

(1) $$\xi_{\mathrm{eff}}=\left(\frac{2e}{\hbar }\right)\left(\frac{M_{s}t_{\mathrm{Co}/\mathrm{Pt}}H_{p}}{J_{Cr_{2}N}}\right)$$

where *M*_s_ and *H*_p_ denote the saturation magnetization of Co/Pt ferromagnetic layer and domain wall depinning field that is defined as *H*_c_ = *H*_p_/cos*θ*, respectively.^[^ ^55^, ^56^ ^]^ Given that the CIMS in the present Hall‐cross structure occurs through the domain nucleation and propagation process, the *H*_p_ value is necessary to estimate *ξ*_eff_ as shown in Equation (1). *H*_p_ was determined by measuring the *H*_c_ as a function of the polar angle (*θ*) with respect to the film surface (see Figure S6 in the Supporting Information). Note that the current‐shunting into [Co/Pt]_3_ layer was excluded from *J*_Cr2N_ by multiplying the ratio of the sheet resistance of the Cr_2_N layer to the entire sheet resistance. It was revealed that *ξ*_eff_ increased monotonically with increasing *t*_Cr2N_ as shown by the red symbols in Figure 4c. To verify this thickness dependence, we examined *ξ*_eff_ by another method, that is a second‐harmonic SOT measurement. Figure 4b1,b2 shows the first‐ and second‐harmonic Hall resistances, respectively, of the sample with *t*_Cr2N_ = 3.6 nm. To distinguish between the damping‐ and field‐like SOTs and various magneto‐thermoelectric components,^[^ ^57^ ^]^ such as the ordinary Nernst effect and anomalous Nernst effect, we conducted the data fitting using the following equation,^[^ ^57^ ^]^

(2) $$R_{xy}^{2\omega }=\frac{R_{\mathrm{AHE}}}{2}\;\frac{H_{\mathrm{DL}}}{\left|H_{x}\right|-H_{k}^{\mathrm{eff}}}+R_{\mathrm{PHE}}\frac{H_{\mathrm{FL}+\mathrm{Oe}}}{\left|H_{x}\right|}+R_{\mathrm{TH}}$$

at the high‐field regime, as shown in Figure 4b2. *R*_AHE_, *R*_PHE_, and *R*_TH_ denote the amplitudes of the anomalous Hall resistance, planar‐Hall resistance obtained by the magnetization rotation on the *xy*‐plane, and the resistance originating from the magneto‐thermoelectric effect, respectively. $H_{k}^{\mathrm{eff}}$ denotes the effective anisotropy field estimated using Equation (S2) in the Supporting Information. *H*_DL_ and *H*_FL+Oe_ are the damping‐like SOT effective field and the superposition of the field‐like effective field and Oersted field, respectively. Using *R*_AHE(PHE)_ ≈ 2.2 Ω (0.02 Ω) and $\mu_{0}H_{k}^{\mathrm{eff}}\approx$ 0.6 T, we obtained *μ*_0_ *H*_DL_ ≈ 4.2 mT and *μ*_0_ *H*_FL+Oe_ ≈ 0.30 mT, resulting in *ξ*_eff_ = *μ*_0_ *H*_DL_/*J*_Cr2N_ ≈ 0.66 mT/MA cm^−2^ for *t*_Cr2N_ = 3.6 nm. Note that *R*_TH_ corresponds to the offset with respect to $R_{xy}^{2\omega }$ ≈ 0 at high field (≈2 T) (Figure 4b2), suggesting that the magneto‐thermoelectric effect can be ruled out from the major origin for CIMS in the present Cr_2_N/[Co/Pt]_3_ system. These results demonstrate that *ξ*_eff_ dependence on *t*_Cr2N_ via second‐harmonic measurements was in agreement with that obtained via CIMS, as shown in Figure 4c. Furthermore, we also evaluated the out‐of‐plane and in‐plane SOT via low field second‐harmonic Hall measurements using the sample with different ferromagnetic layer: substrate//Cr_2_N(8.8 nm)/CoFeB(1 nm)/MgO(2 nm) (see Figure S7 in the Supporting Information). The out‐of‐plane and in‐plane *μ*_0_ *H*_DL_/*J*_Cr2N_ was ≈0.18 mT/MA cm^−2^ and 0.039 mT/MA cm^−2^, respectively, indicating the presence of out‐of‐plane SOT based on the Cr_2_N.

**Figure 5:**
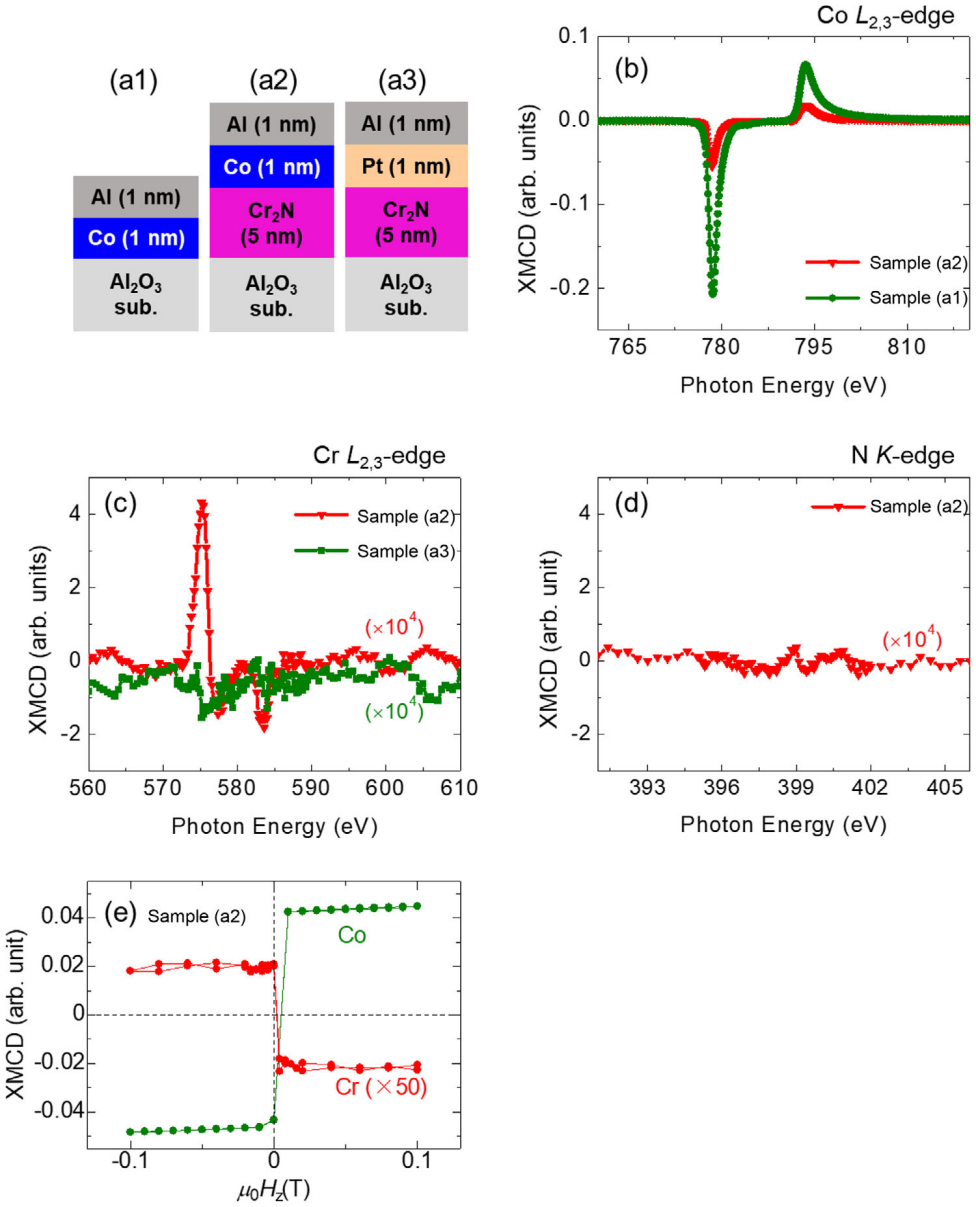
a1–a3) Stacking structures for the XMCD measurement at the synchrotron radiation facility. XMCD spectra for the b) Co *L*_2,3_‐edge), c) Cr *L*_2,3_‐edge, and d) N *K*‐edge of the samples shown in (a1–a3). e) Element‐selective out‐of‐plane magnetic properties for Co and Cr of the sample (a2).

To explore the relationship between the SOT and the interface emergphenomena, we measured the element‐selective magnetic properties at the Cr_2_N/ferromagnetic interfaces by means of X‐ray magnetic circular dichroism (XMCD) for three samples (**Figure** **5**a1,a2): (a1) the pure Co for comparison, (a2) Cr_2_N/Co bilayer, and (a3) Cr_2_N/Pt bilayer. The XMCD signals are shown in Figure 5b–d, and **Table** **1** summarizes spin (*m*_spin_) and orbital (*m*_orb_) magnetic moments of Co, Cr, and N, estimated using the sum rule (see Figure S8 in the Supporting Information).^[^ ^58^, ^59^ ^]^ The resultant values of pure Co (*m*_spin_ ≈ 1.83 *μ*_B_; *m*_orb_ ≈ 0.17 *μ*_B_) were consistent with the calculation results of *m*_spin_ ≈ 1.63 *μ*_B_ and *m*_orb_ ≈ 0.1 *μ*_B_,^[^ ^60^ ^]^ while those of Cr_2_N/Co (*m*_spin_ ≈ 0.99 *μ*_B_; *m*_orb_ ≈ 0.073 *μ*_B_) were smaller when compared to the pure Co. On the other hand, Cr in the Cr_2_N/Co was clearly polarized to be *m*_spin_ ≈ ‐0.063 *μ*_B_ as shown in Figure 5c, which are close to the values for Cr_2_O_3_/Co systems reported.^[^ ^61^ ^]^ Conversely, *m*_spin(orb)_ of Cr in Cr_2_N/Pt was negligible. These results show the presence of $m_{\mathrm{Cr}}^{\mathrm{UC}.}$ originating from the imbalance in the antiferromagnetic structure of Cr_2_N due to the adjacent Co layer, as shown in Figure 1. Regarding N polarization, however, *m*_spin(orb)_ was negligible, as shown in Table 1 and Figure 5d, while N in the Fe–N system shows a finite *m*_spin(orb)_.^[^ ^62^ ^]^ These results confirm the atomic‐layered structure of Cr_2_N, which is terminated by the Cr atomic‐layer at the Cr_2_N/Co interface. To provide further insights into the $m_{\mathrm{Cr}}^{\mathrm{UC}.}$, we measured the element‐selective magnetic hysteresis loops along the *H_z_* direction for Co *L*_3_‐edge and Cr *L*_3_‐edge as shown in Figure 5e. Sharp magnetization switching of Co and Cr was evident at the same *H*_z_, suggesting that the magnetic easy‐axis was aligned in the out‐of‐plane direction. The switching directions were opposite to each other with respect to the magnetic field, indicating antiferromagnetic coupling between Co and Cr, i.e., $m_{\mathrm{Cr}}^{\mathrm{UC}.}$ points down (up) when the magnetization of Co points up (down).

**Table 1:** Spin magnetic moment (*m*_spin_) and orbital magnetic moment (*m*_orb_) for Co, Cr, and N, which are estimated using the sum rule (see Figure S8 in the Supporting Information).^[^ ^58^ ^]^

Sample [*μ*_B_/atom]	Co *m*_spin_ /*m*_orb_	Cr *m*_spin_ /*m*_orb_	N *m*_spin_ /*m*_orb_	Co (calc)^a)^ *m*_spin_/*m*_orb_
Pure Co	1.83 /0.17	N.A.	N.A.	1.63 / ∼0.1
Cr_2_N/Co	0.99 / 0.73	−0.063 / ∼0	∼0 / ∼0	N.A.
Cr_2_N/Pt	N.A.	∼0 / ∼0	∼0 / ∼0	N.A.

^a)^ The values are referred to ref. [59].

**Figure 6:**
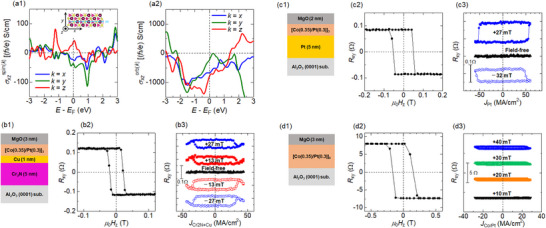
a1,a2) Energy dependent spin‐Hall conductivity ($\sigma_{xz}^{\mathrm{spin}(k)}$) and orbital‐Hall conductivity ($\sigma_{xz}^{\mathrm{orb}(k)}$) for the Cr_2_N, where *x*, *z*, and *k* represent the directions of charge current, spin current, and spin/orbital polarization, respectively. b1–b3,c1–c3,d1–d3) CIMS results for the control Hall‐cross samples, with b1–b3) 1 nm thick Cu layer insertion and parallel charge current to mirror symmetry axis, with c1–c3) Pt underlayer, and d1–d3) with only [Co/Pt]_6_ multilayer.

Hereafter, we discuss possible unconventional SOT mechanisms occurring in the Cr_2_N/[Co/Pt]_3_ system based on theoretical calculations and control samples. The SOT in a ferromagnetic layer generally originates from the spin current generated not only at the bulk part of the spin‐source layer but also at the interface; therefore, both cases are considered individually. First, the spin diffusion length of pure Cr ($\lambda_{s}^{\mathrm{Cr}}$) is reported as∼2.1 nm at RT and ∼4.5 nm at 4.2 K.^[^ ^63^, ^64^ ^]^ Assuming $\lambda_{s}^{\mathrm{Cr}2N}\leq \lambda_{s}^{\mathrm{Cr}}$, due to high atomic density in Cr_2_N comparing to the pure Cr,^[^ ^63^ ^]^ the *ξ*_eff_ is expected to decrease for the thickness *t*_Cr2N_ > $\lambda_{s}^{\mathrm{Cr}2N}$, if the spin current predominantly flows though the Cr_2_N layer. Given this hypothesis is not applicable, as indicated in Figure 4c, it is inferred that the spin current originating from the conventional spin‐Hall effect at the bulk part of the Cr_2_N layer would be a minor cause. Instead, we must consider the long‐range transport property that gives rise to an enhanced *ξ*_eff_ for thicker *t*_Cr2N_. Specifically, the OHE,^[^ ^65^ ^]^ which is reported to emerge in the light elements with weak SOI, has longer orbital diffusion length ($\lambda_{o}^{\mathrm{Cr}}\approx$ 6.1 nm) comparing to the $\lambda_{s}^{\mathrm{Cr}}$.^[^ ^66^ ^]^ Furthermore, enhanced *ξ*_eff_ by increasing the Cr thickness in the Co/Cr system has been reported by another group, which has been explained by the OHE.^[^ ^66^, ^67^ ^]^ In addition to such experimental results, we calculated the intrinsic spin‐Hall conductivity ($\sigma_{xz}^{\mathrm{spin}(k)}$) and orbital‐Hall conductivity ($\sigma_{xz}^{\mathrm{orb}(k)}$) in the Cr_2_N (**Figure** **6**a1,a2) to enable the quantitative comparison between the SHE and OHE contributions in the Cr_2_N, where *x*, *z*, and *k = {x, y, z}* represent the directions of charge current, spin current, and spin/orbital polarization, respectively. Note that we focused only on the possible spin/orbital current flowing in *z*‐direction, which contributes to CIMS of ferromagnetic layers. In addition, other components are summarized in Figure S9 in the Supporting Information. Overall, $\sigma_{xz}^{\mathrm{spin}(k)}$ was one or two orders of magnitude smaller than $\sigma_{xz}^{\mathrm{orb}(k)}$, regardless of *k* direction. Therefore, the hypothesis of the dominant OHE that drown out from the experiments in Figure 4c can be supported by the theoretical prediction, which is similar to the case of pure Cr: *σ* ^spin^ ≈ ‐100 (ℏ/*e*)(S cm^−1^) and *σ* ^orb^ ≈ 8000 (ℏ/*e*)(S cm^−1^).^[^ ^68^ ^]^ Focusing on the *k*‐dependence of OHE, note that we find $\sigma_{xz}^{\mathrm{orb}(z)}>\sigma_{xz}^{\mathrm{orb}(x)}>\sigma_{xz}^{\mathrm{orb}(y)}$ at the Fermi level as shown in Figure 6a2. This implies that the orbital current with *z*‐polarization (*k* = *z*) emerges in the bulk part of Cr_2_N in principle, which is converted into the spin current, resulting in the out‐of‐plane SOT for the field‐free CIMS. To provide insight into the spin and orbital Hall conductivities, the spin and orbital Berry curvatures are analyzed as shown in Figure S10 in the Supporting Information, together with the orbital projected band dispersion of Cr in Cr_2_N for the high‐symmetry line. It was revealed that the *Γ*‐*K* symmetry line of the Cr_2_N MXene dominates the contribution to the spin and orbital Hall conductivity. These characteristics are different from the conventional heavy metals, for example, high Berry curvature near the *X* and *L* points, and near the *P* point and along the path from *H* point contribute to the significant spin‐Hall conductivity of the fcc Pt, and *α*‐Ta, respectively.^[^ ^69^ ^]^ The spin Hall conductivity at the Fermi Energy was ∼2200 (ℏ/*e*)(S cm^−1^) for the fcc Pt and ∼‐142 (ℏ/*e*)(S cm^−1^) for the *α*‐Ta.^[^ ^69^ ^]^ $\sigma_{xz}^{\mathrm{orb}(k)}$ for the Cr_2_N MXene was comparable to the value for the fcc Pt, while $\sigma_{xz}^{\mathrm{spin}(k)}$ was much smaller than those for the fcc Pt and the *α*‐Ta.

Next, it is essential to consider the interfacial contribution to the out‐of‐plane SOT, which can dominantly contribute to the field‐free CIMS. Based on the XMCD results, we identified the $m_{\mathrm{Cr}}^{\mathrm{UC}.}$ oriented in the out‐of‐plane direction at the interface owing to neighboring Co. Even though the conversion efficiency from orbital to spin might not be strong in Cr_2_N, as in the case of pure Cr,^[^ ^66^ ^]^ the spin would be scattered or transferred depending on the direction of $m_{\mathrm{Cr}}^{\mathrm{UC}.}$, which is likely a spin‐filtering effect by the $m_{\mathrm{Cr}}^{\mathrm{UC}.}$ at interface.^[^ ^27^, ^47^, ^69^ ^]^ Because of the antiferromagnetic coupling between the *M*_Co/Pt_ and the $m_{\mathrm{Cr}}^{\mathrm{UC}.}$, the polarized spin transferred through the interface is always opposite to the *M*_Co/Pt_, resulting in field‐free deterministic CIMS. To validate this, we examined the CIMS properties of the control sample with the 1‐nm‐thick Cu insertion between the Cr_2_N layer and the [Co/Pt]_3_ ferromagnetic multilayer, i.e., substrate//Cr_2_N(5 nm)/Cu(1 nm)/[Co(0.35 nm)/Pt(0.3 nm)]_3_/MgO(3 nm) (Figure 6b1). Due to weak SOI and non‐magnetism of Cu, which can identify the effect of $m_{\mathrm{Cr}}^{\mathrm{UC}.}$ on the observed field‐free CIMS. Figure 6b2,b3 shows the representative out‐of‐plane AHE and the CIMS, respectively, where the charge current direction is parallel to the mirror symmetry (*m* || *I*). Note that field‐dependent CIMS was observed with the same polarity as the main sample (Figure 2c3), while no field‐free CIMS was evident by the Cu insertion. The result was comparable to the different configuration with the orthogonal charge current to the mirror symmetry axis as well (see Figure S11 in the Supporting Information), which can be attributed to the absence of induced magnetic moment of Cu. We thus conclude that magnetic moment of Cr induced by the Co at the interface plays an essential role for field‐free CIMS, which may become a key to elucidate one of the possible scenarios of the spin‐filtering mechanism at the interface between MXene/ferromagnetic layer.

Some reports show that SOT‐devices with Co/Pt multilayer exhibit CIMS by itself, without non‐magnetic spin sources, which is referred to as self‐induced SOT.^[^ ^70^ ^]^ However, the self‐induced SOT cannot become a major origin for the field‐free CIMS in the present Cr_2_N/[Co/Pt]_3_ due to the following considerations. We examined the CIMS properties of the other control samples by replacing the Cr_2_N layer with a Pt layer, as shown in Figure 6c1–c3. Unlike the results for the main Cr_2_N/[Co/Pt]_3_, the polarity of CIMS was reversed: CW (CCW) for negative (positive) *H_x_*, and no field‐free CIMS was observed, although the [Co/Pt]_3_ multilayer is consistent. This CIMS property is consistent with that observed in the conventional SOT‐device such as Pt/CoPt bilayer systems, in which *y*‐polarized spin current dominates the CIMS mechanisms.^[^ ^57^ ^]^ These results suggest that the impact of spin source layer on SOT is much greater than that of Co/Pt multilayer itself. Furthermore, we confirmed the absence of field‐free CIMS in the controlled [Co/Pt]_6_ sample without Cr_2_N layer as shown in Figure 6d1–d3. We thus infer again that the impact of [Co/Pt]_3_ for the field‐free CIMS is minor, if any.

An interlayer exchange interaction owing to the $m_{\mathrm{Cr}}^{\mathrm{UC}.}$ cannot contribute to the field‐free CIMS, in terms of the collinear magnetic structure of $m_{\mathrm{Cr}}^{\mathrm{UC}.}$ and Co. It has been reported that the field‐free CIMS can be observed in an SOT bilayer structure consisting of an antiferromagnetic layer with an in‐plane Néel ventor and a perpendicularly magnetized ferromagnetic layer.^[^ ^28^ ^]^ This is because the magnetic structure of ferromagnetic layer near the interface can be tilted to the in‐plane direction through the interlayer exchange interaction, resulting in the effective in‐plane magnetic field to break the inversion symmetry. Conversely, the Cr_2_N has collinear antiferromagnetic structures with out‐of‐plane Néel ventors (Figure 2a), which couples with perpendicularly magnetized Co/Pt ferromagnetic layer and the resultant magnetic structure cannot break the inversion symmetry. Therefore, field‐free CIMS cannot be explained by the mechanism of interlayer exchange interaction for MXene‐based SOT‐devices.

It should be noted that non‐centrosymmetric materials, such as monolayer TMDCs, exert out‐of‐plane SOTs in the adjacent ferromagnetic layer when the charge current flows orthogonal to the mirror axis, in which the sign of the out‐of‐plane SOT reverses with the sign of the charge current direction.^[^ ^33^, ^34^, ^36^ ^]^ Recent theoretical study predicts a momentum‐independent uniform spin configuration known as persistent spin texture for the other 2D non‐centrosymmetric materials of CdTe and ZnTe,^[^ ^71^ ^]^ which is expected to realize spintronic devices because of many advantages such as robustness against strain, layer thickness, and crystal distortion. In addition, the mechanism of the OHE that occurs in non‐centrosymmetric materials has recently been examined as an intrinsic property.^[^ ^72^ ^]^ All of these are classified into the symmetry‐driven intrinsic mechanisms. In contrast to that, an unconventional out‐of‐plane SOT can emerge by the charge current flowing even in the direction parallel to the mirror axis, which is the specific characteristics of MXenes that cannot be explained by such the conventional scenario with inversion symmetry mentioned above. Therefore, investigation of the 2D‐MXene/ferromagnet interface could be a key to facilitate the field‐free CIMS in the SOT‐device with the 2D‐MXene.

The 2D‐MXene has many advantages in principle, such as the bottom‐up formability by the conventional sputtering, the phase stability, the sustainable light elements, and the process compatibility with CMOS technology. Furthermore, the charge‐current‐direction independent unconventional out‐of‐plane SOT may lead to a robust field‐free CIMS for 2D SOT‐MRAMs in the future.

## 3 Conclusion

We have reported the first demonstration of CIMS in the SOT‐device with the sputter‐deposited bare 2D‐MXene of Cr_2_N: substrate/Cr_2_N/[Co/Pt]_3_/MgO cap. The specific characteristics of the MXene‐based SOT‐device is the field‐free CIMS via a possible out‐of‐plane unconventional SOT, regardless of the in‐plane charge current direction with respect to crystal symmetry of Cr_2_N, which is likely a robust field‐free CIMS by MXene. A critical current density is ∼10^7^ A cm^−2^, which is comparable to that of the conventional heavy‐metal/ferromagnet systems. The Cr_2_N thickness dependence of SOT efficiency indicates the bulk OHE contribution, and $\sigma_{xz}^{\mathrm{orb}(z)}$ dominates the OHE in Cr_2_N based on the first‐principles calculations. The XMCD study indicates that the Cr of Cr_2_N is polarized by the adjacent ferromagnetic Co of Co/Pt multilayer, and the $m_{\mathrm{Cr}}^{\mathrm{UC}.}$ antiferromagnetically couples to the magnetic moment of Co. Thus, the field‐free CIMS observed in MXene‐based SOT‐device can be predominantly attributed to the spin with *z* ‐component originating from the bulk OHE and the spin‐filtering‐like effect at the interface due to $m_{\mathrm{Cr}}^{\mathrm{UC}.}$.

## 4 Experimental Section

### Film Fabrication and Characterization

An Al_2_O_3_ crystal substrate with a (0001) plane orientation was cleaned with ethanol and acetone via ultrasonic cleaning and flash annealed at 650 °C for 30 min in the sputtering chamber with a base vacuum pressure of approximately 10^−7^ Pa. The Cr_2_N film was deposited on the substrate using the DC magnetron reactive sputtering for the Cr target at *T*_sub_ from RT to 650 °C with the gas mixture of N_2_/(Ar + N_2_) = 5%, where the deposition rate was 1.68 nm min^−1^. The Co/Pt multilayer and MgO capping layer were deposited via DC and RF magnetron sputtering at RT. The crystal structure was investigated via X‐ray diffraction (XRD; SmartLab; Rigaku Corporation) with Cu‐*K*_α_ radiation. The surface roughness is evaluated via atomic force microscopy. The magnetic properties and anomalous Hall effect were measured at RT using a magnetic property measurement system (MPMS; Quantum Design Inc.) and a physical property measurement system (Dynacool; Quantum Design Inc.), respectively.

### Element Selective Magnetic Properties Measured via XMCD

The XMCD measurements were performed at the BL14U Synchrotron Radiation Facility, NanoTerasu. Soft X‐ray absorption spectra (XAS) were recorded using the total electron yield (TEY) method while scanning photon energy at RT. The XMCD signal was obtained by subtracting each XAS signal for circularly polarized light with positive and negative helicities. In particular, for Cr and N with tiny magnetic moments, the XAS measurement for each helicity was repeated five times and averaged to boost the signal‐to‐noise ratio. The magnetic field was applied perpendicularly to the surface of the sample. Element‐selective magnetic properties against the applied field (ESMH) were measured for the *L*_3_‐edge of Co and Cr at RT.

### CIMS and Second‐Harmonic Measurements

Photolithography and Ar ion milling were employed to fabricate the measurement devices with Hall cross and pillar patterns, in which the line width of charge current channel is 10 µm and the diameter of pillar is ∼7 µm. A customized system was used for the CIMS experiments. A rectangular current pulse was applied to the current channel of the Hall cross devices with durations of 10 ms using a pulse generator (FG420; Yokogawa Electric Co.). The Hall voltage was recorded using a digital multimeter (7555; Yokogawa Electric Co.) at every interval between the current pulses, that is, 1 s after the last current pulse. The DC current to sense the Hall voltage was 0.5 mA (≈0.60 MA cm^−2^) for Hall cross devices, and 0.2 mA (≈0.14 MA cm^−2^) for pillar devices, which were applied using a DC power source (G210, Yokogawa Electric Co.). The sensing current density was approximately 2% of critical current density, which can be negligibly small for CIMS. The magnetic field from the electromagnet was uniform within a gap length of 3 cm and an area 5 cm in diameter. The device was placed away from the electromagnet for field‐free CIMS to eliminate any residual field from the magnetic pole pieces. The second‐harmonic Hall voltage was recorded using a lock‐in amplifier (LI5640, NF Co.) while the in‐plane applied field was scanned. A sinusoidal wave with an effective amplitude of 3 mA (≈4 MA cm^−2^) and frequency of 33.123 Hz was applied using a pulse generator (FG420; Yokogawa Electric Co.). A common device and sample package were used for CIMS and second‐harmonic measurements. All measurements were performed at RT.

### Computational Procedure for Spin/Orbital‐Hall Conductivities

First‐principles calculations were performed using the Vienna ab initio Simulation Package.^[^ ^73^ ^]^ Projector‐augmented wave (PAW) pseudo‐potentials were used for the atomic potentials of Cr and N with a plane‐wave cut‐off energy of 500 eV.^[^ ^74^ ^]^ The generalized gradient approximation for the exchange and correlation energies were adopted, including the spin–orbit interaction with 10 × 10 × 10 k‐points in the first Brillouin zone.^[^ ^75^ ^]^ The onsite Coulomb interaction was considered, *U* = 3 eV, for the Cr atom. The lattice parameters of Cr_2_N are the same as those shown in Figure 2a.^[^ ^76^ ^]^ The spin‐Hall conductivity (*σ* ^spin^) and orbital‐Hall conductivity (*σ* ^orb^) were calculated based on linear response theory as:^[^ ^77^, ^78^ ^]^

(3) $$\sigma_{\alpha \beta }^{X\left(\gamma \right)}\left(E\right)=\frac{e}{V}\sum_{k}\Omega_{\alpha \beta }^{X\left(\gamma \right)}\left(k,E\right)$$

$\Omega_{\alpha \beta }^{X(\gamma )}\left(k,E\right)$ is the orbital Berry curvature provided by:^[^ ^79^ ^]^

(4) ΩαβXγk,E=2ℏ2me2∑n>mfkmE−fknEImkmp^αXγknknp^βkmεkn−εkm2

where *V* denotes the unit‐cell volume, *m_e_* denotes the electron mass, *m* and *n* denote the occupied and unoccupied band indices, respectively. ${\hat{p}}_{\beta }^{X(\gamma )}$ denotes “spin” or “orbital” current operator (X = spin or orbital), where ${\hat{p}}_{\alpha }^{\mathrm{spin}(\gamma )}={\hat{p}}_{\alpha }\;{\hat{s}}_{\gamma }+{\hat{s}}_{\gamma }{\hat{p}}_{\alpha }$ and ${\hat{p}}_{\alpha }^{\mathrm{orbital}(\gamma )}={\hat{p}}_{\alpha }\;{\hat{L}}_{\gamma }+L_{\gamma }{\hat{p}}_{\alpha }$. Furthermore, ${\hat{p}}_{\alpha }$ (${\hat{p}}_{\beta }$) is *α*(*β*)‐axis component of the momentum operator, ${\hat{s}}_{\gamma }$ denotes the spin angular momentum operator with the spin quantum axis along γ direction, and ${\hat{L}}_{\gamma }$ denotes the orbital angular momentum operator along γ direction. Additionally, |**k** *n*〉 denotes the eigenstate with the eigenenergy ε_**k** *n*_, and *f*_**k** *n*_(*E*) denotes the occupation function for band *n* and wave‐vector **k** at the energy (*E*) relative to the Fermi level (*E*_F_). The *σ* ^spin(orb)^ of Cr_2_N was computed using 30 × 30 × 30 k points in the first Brillouin zone.

## Conflict of Interest

The authors declare no conflict of interest.

## Supporting information



Supporting Information

## Data Availability

The data that support the findings of this study are available from the corresponding author upon reasonable request.
