# Supplementary material for: Unconventional Spin–Orbit Torques by 2D Multilayered MXenes for Future Nonvolatile Magnetic Memories
Source: Small. 2025 May 15;21(25):2500626. doi: 10.1002/smll.202500626 (PMC12199109; doi:10.1002/smll.202500626)
Supplement: Supplementary file 1 — Supporting Information [file SMLL-21-2500626-s001.pdf]

NANO · MICRO
small

Supporting Information

for *Small*, DOI 10.1002/smll.202500626

Unconventional Spin–Orbit Torques by 2D Multilayered MXenes for Future Nonvolatile Magnetic Memories

*Prabhat Kumar, Yoshio Miura, Yoshinori Kotani, Akiho Sumiyoshiya, Tetsuya Nakamura, Gaurav K. Shukla and Shinji Isogami**

Supporting Information

Unconventional spin-orbit torques by two-dimensional multilayered MXenes for future nonvolatile magnetic memories

Prabhat Kumar,[1] *Yoshio Miura,*[1,2] *Yoshinori Kotani,*[3] *Akiho Sumiyoshiya,*[3] *Tetsuya Nakamura,*[3,4] *Gaurav Kumar Shukla,*[1] *and Shinji Isogami* [1]*

[1] Research Center for Magnetic and Spintronic Materials,
National Institute for Materials Science (NIMS).
Sengen 1-2-1, Tsukuba, Ibaraki, 305-0047, JAPAN.

[2] Faculty of Electrical Engineering and Electronics,
Kyoto Institute of Technology.
Hashikami-cho, Matsugasaki, Sakyo-ku, Kyoto, 606-8585, JAPAN

[3] Photon Science Innovation Center (PhoSIC),
Aoba 468-1, Aramaki-Aza, Aoba, Sendai, 980-8572, JAPAN.

[4] International Center for Synchrotron Radiation Innovation Smart Center (SRIS),
Tohoku University.
Aoba 468-1, Aramaki-Aza, Aoba, Sendai, 980-8572, JAPAN.

*E-mail : isogami.shinji@nims.go.jp

Table of Contents

§S1. Magnetic properties for the SOT stacking film:

§S2. Resistivity of the Cr_2N layer and [Co/Pt] ferromagnetic multilayer:

§S3. SOT-device deposited at low temperature:

§S4. Field-free CIMS loops for different Hall-bar devices:

§S5. Magnetization switching by field in microfabricated pillar devices:

§S6. Estimation of the pinning field of [Co/Pt] ferromagnetic multilayer:

§S7. Estimation of out-of-plane damping-like field via second-harmonic Hall measurements with Cr_2N/CoFeB SOT-device:

§S8. Estimation of spin and orbital moment via Sum rule:

§S9. Energy dependent spin/orbital Hall conductivity via first-principles calculation:

§S10. Spin/orbital Hall conductivity-projected band structures and Berry curvatures via first-principles calculation:

§S11. CIMS for SOT-device with 1-nm-Cu insertion, $Cr_2N/Cu/[Co/Pt]_3$:

§S1. Magnetic properties for the SOT stacking film:

Figure S1 shows the magnetic properties for the Co/Pt multilayer with three periods [Co(0.35 nm)/Pt(0.3 nm)]$_3$, as a function of external magnetic field (*M-H* loops). Saturation field was $\mu_0 H \approx 0.5$ T for the in-plane *M-H* loops (shown by black symbols), while $\mu_0 H \approx 0$ T for the out-of-plane *M-H* loops (shown by red symbols), suggesting that the magnetic easy-axis points in the out-of-plane direction. The uniaxial magnetic anisotropy energy density (K_u) is given by,

$$K_u = K_u^{eff} + \frac{\mu_0 M_s^2}{2}, \quad \text{(S1)}$$

$$K_u^{eff} = \left(\mu_0 \int_0^{M_s} H\, dm\right)_{hard-axis} - \left(\mu_0 \int_0^{M_s} H\, dm\right)_{easy-axis}, \quad \text{(S2)}$$

where the second term of Eq. (S1) represents the demagnetization component. The K_u was estimated to be 0.42 MJ/m^3, which agrees with the value reported by another group.[S1] Note that the K_u for Co/Pt multilayer system can be modulated dominantly by the Co thickness.[S1]

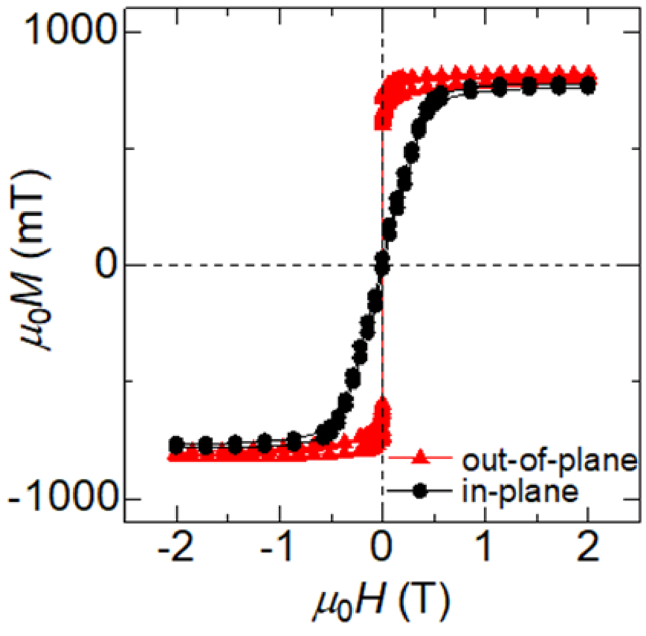

Fig. S1. Magnetic hysteresis loops (*M-H* loops) of the SOT stacking sample, Al_2O_3 substrate//Cr_2N(5 nm)/[Co(0.35 nm)/Pt(0.3 nm)]$_3$/MgO(2 nm), where the magnetic field was swept along both in-plane and out-of-plane directions.

§S2. Resistivity of the Cr_2N layer and [Co/Pt] ferromagnetic multilayer:

In order to determine the resistivity of the Cr_2N (ρ_{Cr2N}) and $[Co/Pt]_3$ ($\rho_{Co/Pt}$) layers, the sheet conductance [$R_{xx}^{-1}(L/W)$] of the current channel with the length (L = 25 μm) and width (W = 10 μm) in the Hall-cross structure was plotted against the Cr_2N film thickness (t_{Cr2N}) as shown in Fig. S2. The stacking structure for this measurement is substrate//Cr_2N(t_{Cr2N})/[Co(0.35 nm)/Pt(0.3 nm)]$_3$/MgO(2 nm), which is the same sample as that for CIMS measurements in Fig. 3 (main text). The data plot was fitted by a linear function as,

$$R_{xx}^{-1}\left(\frac{L}{W}\right) = \frac{t_{\mathrm{Cr_2N}}}{\rho_{\mathrm{Cr_2N}}} + \frac{t_{\mathrm{Co/Pt}}}{\rho_{\mathrm{Co/Pt}}} \quad , \qquad \text{(S3)}$$

where the t_{Cr2N} was varied from 1 nm to 9 nm. The slope and the intercept of the fitting line correspond to the ρ_{Cr2N} and $\rho_{Co/Pt}$, respectively. The linear fit was succeeded as shown by red line of Fig. S2, resulting in $\rho_{Cr2N} \approx 73$ μΩ cm and $\rho_{Co/Pt} \approx 136$ μΩ cm. The ρ_{Cr2N} was in agreement with another report.[S2]

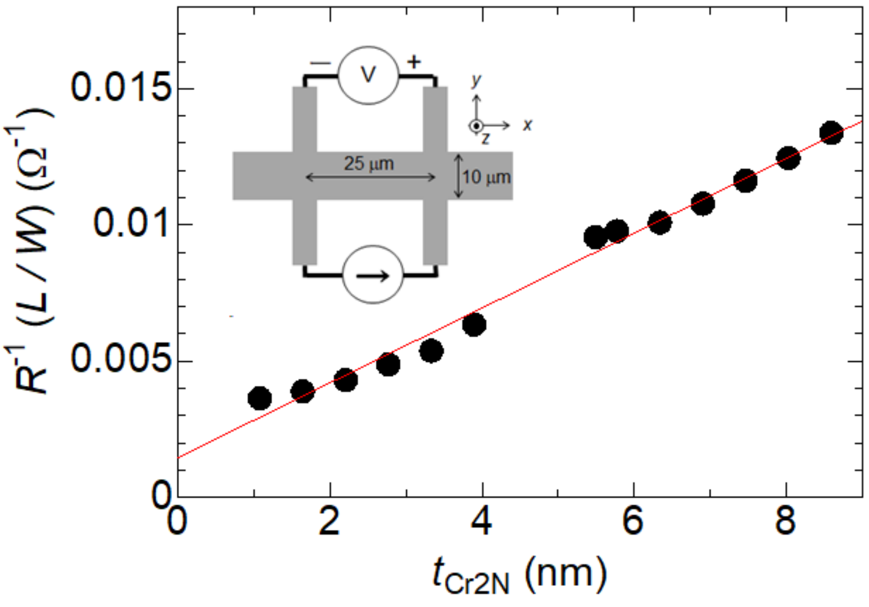

Fig. S2. Cr_2N layer thickness (t_{Cr2N}) dependence of the entire sheet conductance [$R_{xx}^{-1}(L/W)$] for the Hall-cross structure with the stacking of substrate//Cr_2N(t_{Cr2N})/[Co(0.35 nm)/Pt(0.3 nm)]$_3$/MgO(2 nm). The red line represents the fitting results using Eq. (S3) with $\rho_{Cr2N} \approx 73$ μΩ cm, $\rho_{Co/Pt} \approx 136$ μΩ cm, and $t_{Co/Pt}$ = 1.9 nm.

§S3. SOT-device deposited at low temperature:

Figure 3(c5) in the main text shows the J_{Cr2N}-H_x diagrams for the SOT-deices, of which Cr_2N MXenes were deposited at the substrate temperatures of 350 °C and 650 °C. The raw data for 650 °C are shown in main text, while Figs. S3(a)-S3(c) show the raw data for 350 °C. Both AHE loops are similar to those for 650 °C. In addition, we confirmed the same polarity of CIMS as that for 650 °C, that is, CW (CCW) for positive (negative) H_x, even in the field-free CIMS. Therefore, the device fabricated at low temperature works as that done at high temperature, which would be an advantage in terms of the compatibility with CMOS technology.

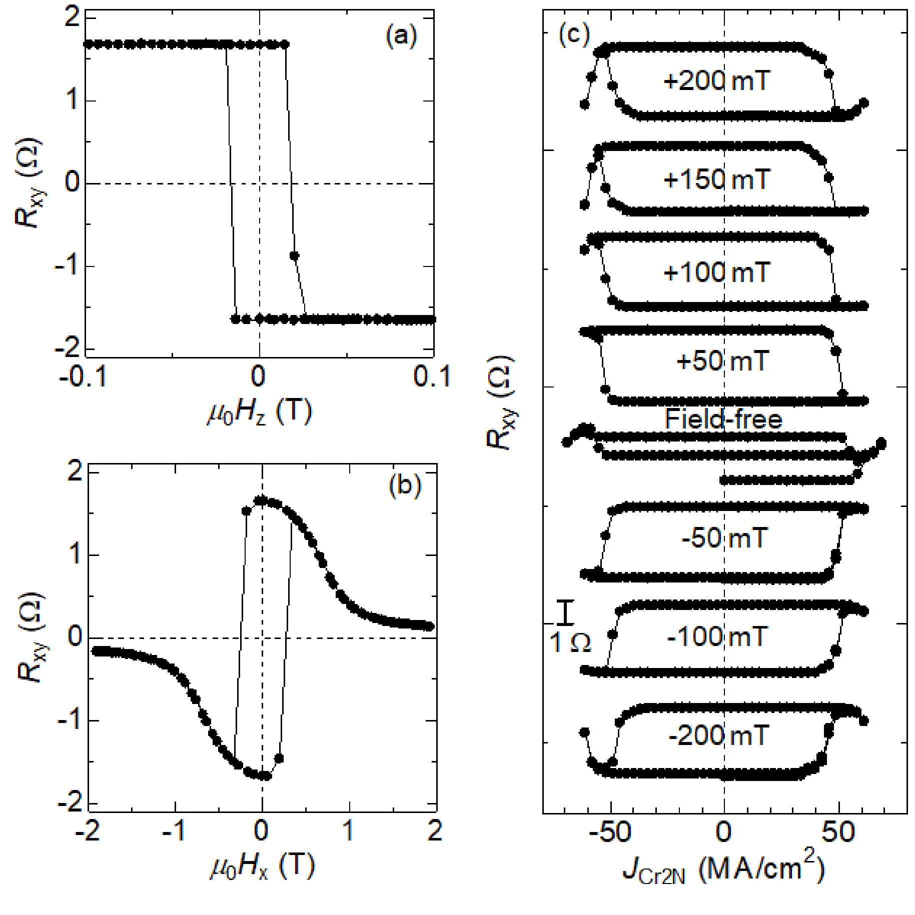

Fig. S3. Hysteresis loops of out-of-plane AHE (a), in-plane AHE (b), and field dependent CIMS (c) for the Hall-cross sample: substrate//Cr_2N(5 nm)/[Co(0.35 nm)/Pt(0.3 nm)]$_3$/MgO(2 nm). Substrate temperature while the Cr_2N deposition is 350 °C, and the charge current direction is prrallel to the mirror line of crystal symmetry, as shown in Fig. 3(b1) in the main text.

§S4. Field-free CIMS loops for different devices:

Many Hall bar SOT-devices were microfabricated on one sample [Fig. S4(a1)]. The sufficient perpendicular magnetic anisotropy was observed [Figs. S4(a2) and S4(a3)]. CIMS measurements were carried out with $\mu_0 H_x$ = +29 mT for the 10~20 devices to confirm reproducibility and deviation of field-free CIMS loops. Figure S4(a4) shows the representative field-free CIMS loops among the measured devices, starting from the magnetization state of up (red) and down (blue) directions. The partial CIMS was confirmed for all devices and the polarity of CIMS was clockwise regardless of the initial magnetization state, which are consistent with the result in Fig. 3(c4) in the main text.

Figures S4(b1)-S4(b4) show the SOT-device with 1-nm-thick Pt layer insertion. Resultant CIMS polarity was reversed comparing to the main sample without Pt insertion. Although the field-free CIMS was appeared, of which amplitude ratio relative to the entire CIMS was still ~15 %. These results show the superposition of the spin current in the Pt layer and the *z*-polarized spin current converted from the pronounced *z*-polarized orbital current in the bulk part of the Cr_2N layer.

Figures S4(c1)-S4(c4) show the SOT-device with 1-nm-thick Cr layer insertion. Resultant CIMS polarity was the same as the main sample without Cr insertion, i.e., CW (CCW) for positive (negative) H_x. Moreover, the ratio of field-free CIMS was enhanced comparing to that of the main sample, as plotted in Fig. 2(f) in the main text. This can be attributed to the enhancement of *z*-polarized spin component by the $m_{\mathrm{Cr}}^{\mathrm{UC.}}$ that is emerged at the Cr/[Co/Pt]$_3$ interface. We thus infer that the interfacial spin-filtering-like effect due to $m_{\mathrm{Cr}}^{\mathrm{UC.}}$ can be an universal property for the out-of-plane SOT in addition to the OHE in the bulk mentioned in the main text, which plays a crucial role in field-free CIMS in MXene-based SOT-devices.

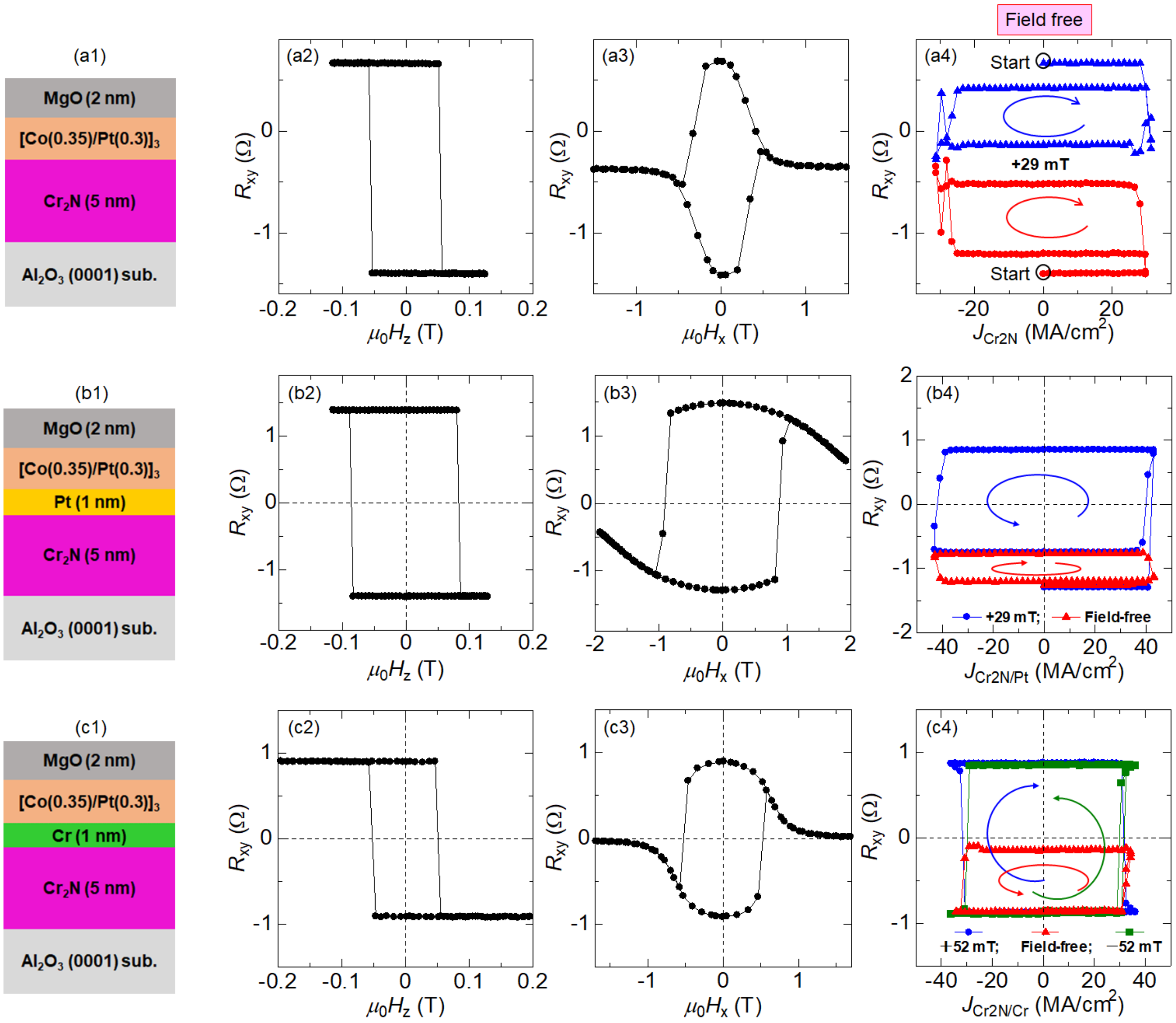

Fig. S4. (a) Out-of-plane (a2), in-plane (b3), and CIMS loops with $\mu_0 H_x$ = +29 mT, which starts from two different initial magnetic states for the Hall-cross sample (a1). (b,c) Same as (a) but for the samples (b1) and (c1). Note that all the charge current directions are parallel to the mirror symmetry axes ($m \parallel I$).

§S5. Magnetization switching by field in microfabricated pillar devices:

The amplitude of field-free CIMS in Hall-cross geometry showed ~20% of the amplitude of full switching by field. This can be attributed to the multidomain nucleation while CIMS, so that we conducted the microfabrication of SOT-device with pillar ferromagnet. Figure S5(a) shows the polar-Kerr microscopy image before and after magnetization switching of the pillar device by magnetic fields. Abrupt changes in Kerr signal appeared, and an edge-domain was not observed while the field switching. The pillar device could be responsible for the 94% field-free CIMS, as shown in Fig. 3(c6) in the main text. Figure S5(b) shows all results of a pillar device with two different in-plane charge current directions with respect to the crystal mirror symmetry.

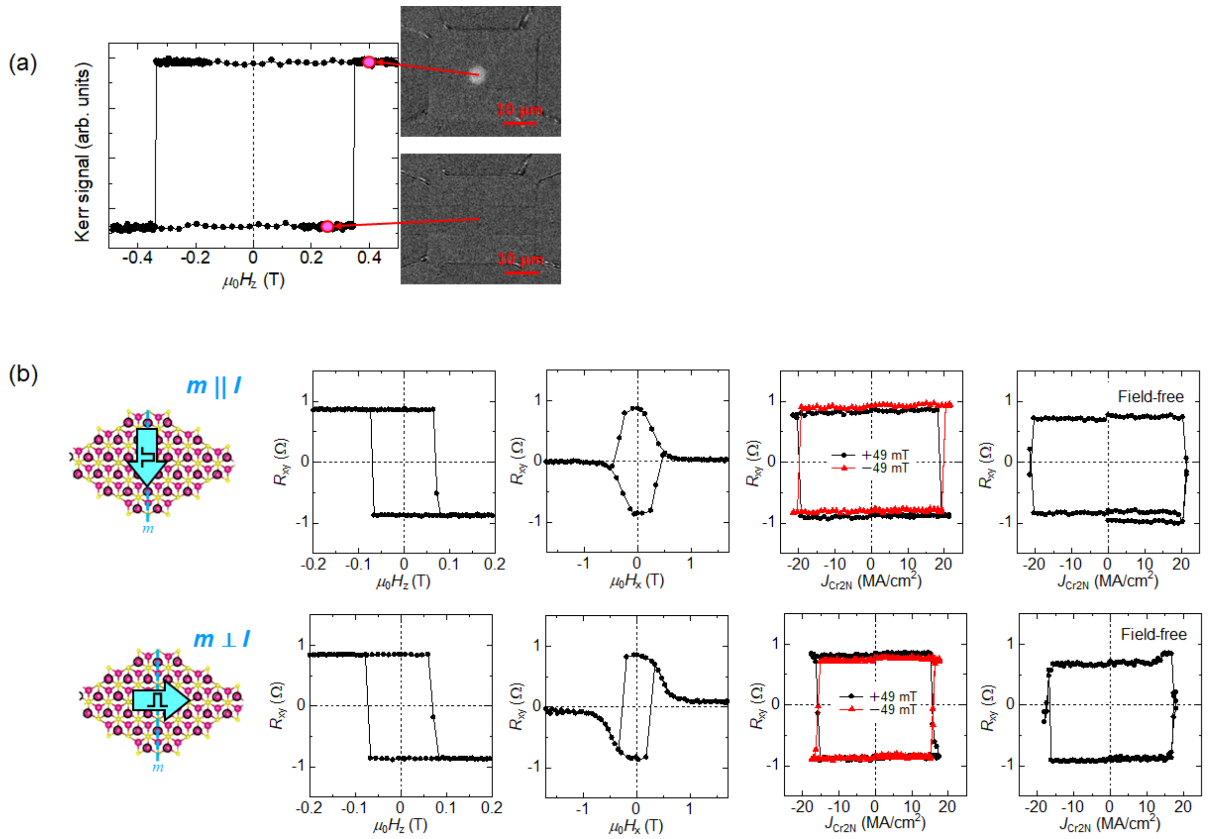

Fig. S5. (a) Kerr hysteresis loop and polar Kerr microscopy images for the SOT-device with pillar ferromagnet with ~7 μm in diameter. (b) Out-of-plane AHE, in-plane AHE, and CIMS with and without field for another device, but with the same pillar size.

§S6. Estimation of the pinning field of [Co/Pt] ferromagnetic multilayer:

In the Hall-cross SOT-device with large ferromagnetic area, the present CIMS occurs through the domain nucleation first, followed by the domain-wall propagation.[S3] Because the effective SOT efficiency (ξ_{eff}) in Eq. (2) in the main text is governed by the depinning field (H_p) of domain-walls, we determined the H_p based on the angular dependent anomalous Hall loops for each t_{Cr2N} as shown in Figs. S6(a) and S6(b). The magnetic coercivity (H_c) increased with increasing the polar angle (θ), and the plot was fitted using the formula: $H_c = H_p/\cos\theta$.[S4] Figure S6(c) summarizes the resultant H_p comparing to the H_c, as a function of t_{Cr2N}. It can be seen that the t_{Cr2N} dependence of H_p agrees with that of H_c, which is in agreement with the results on the other system with Ru thickness dependence.[S4]

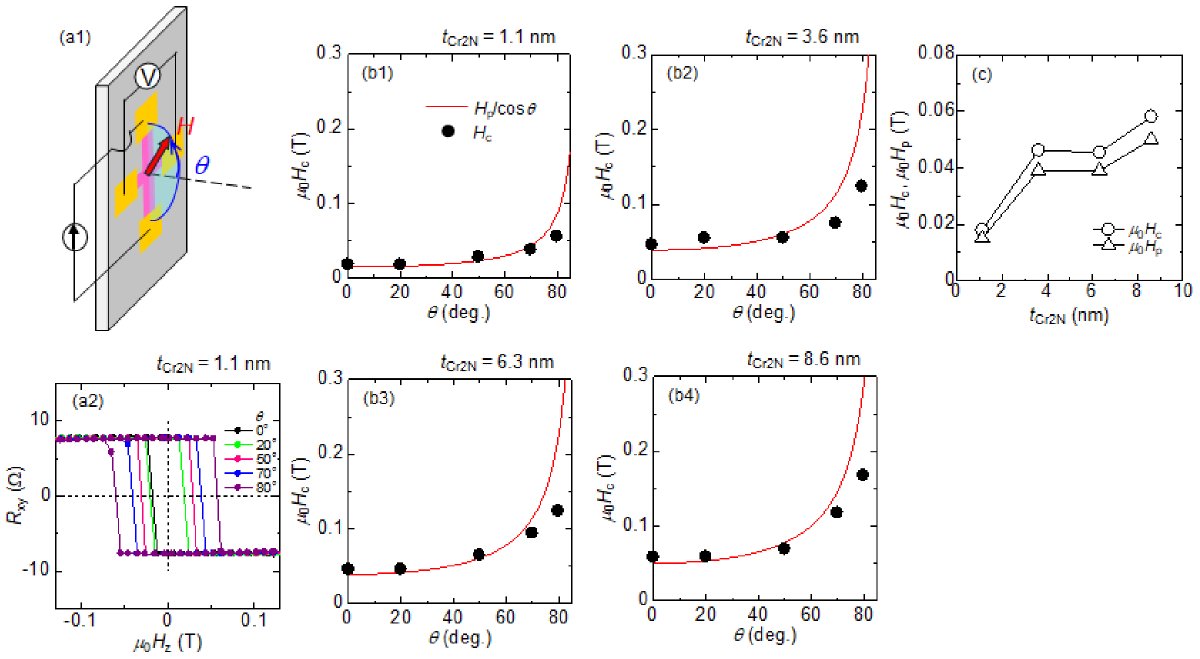

Fig. S6. (a1,a2) Measurement geometry for the polar-angle dependence of anomalous Hall loops. (b1-b4) The H_c values as a function of polar angle (θ) for each Cr_2N layer thickness (t_{Cr2N}) in the sample: substrate//Cr_2N(t_{Cr2N})/[Co(0.35 nm)/Pt(0.3 nm)]$_3$/MgO(2 nm). The solid symbols and red lines represent the measured data and the fitting results using the formula: $H_c = H_p/\cos\theta$, respectively. (c) t_{Cr2N} dependence of H_c and H_p.

§S7. Estimation of out-of-plane damping-like field via low field second-harmonic Hall measurements with Cr_2N/CoFeB SOT-device:

To evaluate the out-of-plane damping-like effective field, second harmonic Hall measurements was conducted with the rotating in-plane external magnetic field ($\mu_0 H_{ext}$ = 50 mT) as shown in the inset of Fig. S7(a) at room temperature, where φ denotes the azimuthal angle of H_{ext}. Figure S7(a) shows the anomalous Hall voltage (V_{xy}) as a function of out-of-plane magnetic field (H_z), indicating that the easy-axis of CoFeB layer does not point in *z*-direction. Figures S7(b) shows the φ-dependent first-harmonic Hall voltage with the AC current density of 2 MA/cm^2, that is, planar Hall effect (PHE). The experimental plots were fitted by the formula: $V_{xy} = A\sin 2\varphi$, where A represents the amplitude of PHE. Figure S7(c) shows the φ-dependent second-harmonic Hall voltage. We found φ-dependent asymmetric behavior, which is due to the superposition of out-of-plane SOT [$\tau_{\perp}(\varphi) = \tau_A \cos(\varphi) + \tau_B$], in addition to the conventional in-plane SOT [$\tau_{\parallel}(\varphi) = \tau_S \cos(\varphi)$], where $\tau_A = m \times \sigma_y$, $\tau_B = m \times (\sigma_z \times m)$, and $\tau_S = m \times (\sigma_y \times m)$. Using the formula suggested by MacNeill *et al.*[S5] with H_{ext} = 50 mT, M_s = 1000 kA/m, V_{AHE} = 0.80 mV, and V_{PHE} = 17.6 μV, data plots were successfully fitted by τ_A/γ = 0.078 mT, τ_B/γ = 0.373 mT, and τ_S/γ = 0.085 mT. We can confirm the presence of out-of-plane SOT by the in-plane charge current flowing in the Cr_2N MXene layer.

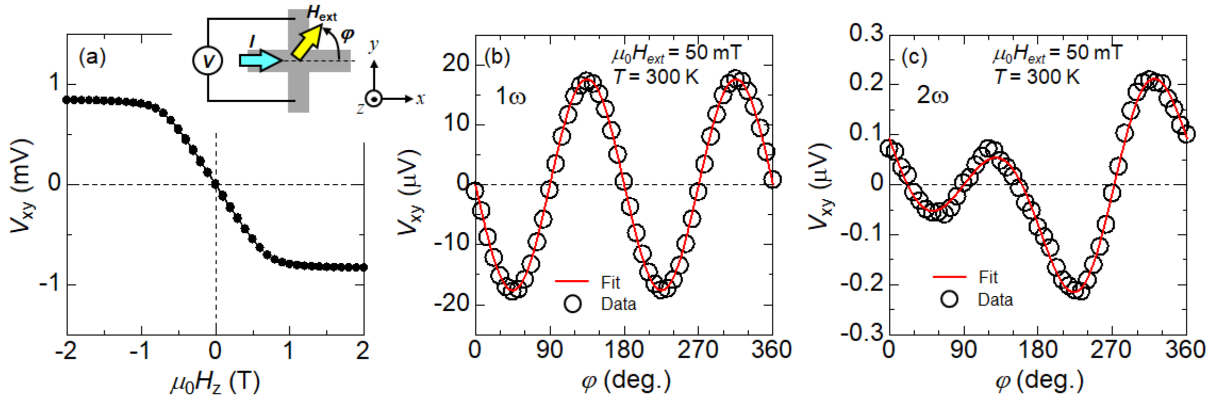

Fig. S7. (a-c) Out-of-plane anomalous Hall hysteresis loop (a), first-harmonic Hall voltage (b), and second-harmonic Hall voltage (c) for the samples, substrate//Cr_2N(8.8 nm)/CoFeB(1 nm)/MgO(3 nm), where in-plane external field (H_x) and measurement temperature were 50 mT and 300 K, respectively.

§S8. Estimation of spin and orbital moment via Sum rule:

X-ray magnetic circular dichroism (XMCD) measurements were performed in total electron yield (TEY) mode by applying an external magnetic field of *H* in *z*-direction, and x-ray absorption spectroscopy (XAS) signal μ was recorded for the positive and negative helicity (σ) of the circularly polarized light. In addition, the XAS signal was also recorded by reversing the polarity of *H* (in *z* direction), which resulted in four XAS spectra, namely μ_H^σ. Post XMCD signal (μ^m) was obtained following equation,

$$\mu^m = \frac{(\mu_{-H}^{+\sigma} - \mu_{-H}^{-\sigma}) - (\mu_{+H}^{+\sigma} - \mu_{+H}^{-\sigma})}{2}. \qquad \text{(S4)}$$

Following the sum rule, spin and orbital magnetic moment, m_{spin} and m_{orb}, respectively, are estimated which can be given by, [S6]

$$m_{\mathrm{spin}} = -\frac{(6p-4q)}{r} n_h, \text{ and } m_{\mathrm{orb}} = -\frac{4q}{3r} n_h, \qquad \text{(S5)}$$

where, the term n_h stands for the number of the core hole that is chosen to be 2.49 for Co,[S7] and 4.45 for Cr.[S8] The quantities *p*, *q*, and *r* were determined after integration of XAS, and corresponding XMCD spectra, as shown in Fig. S8.

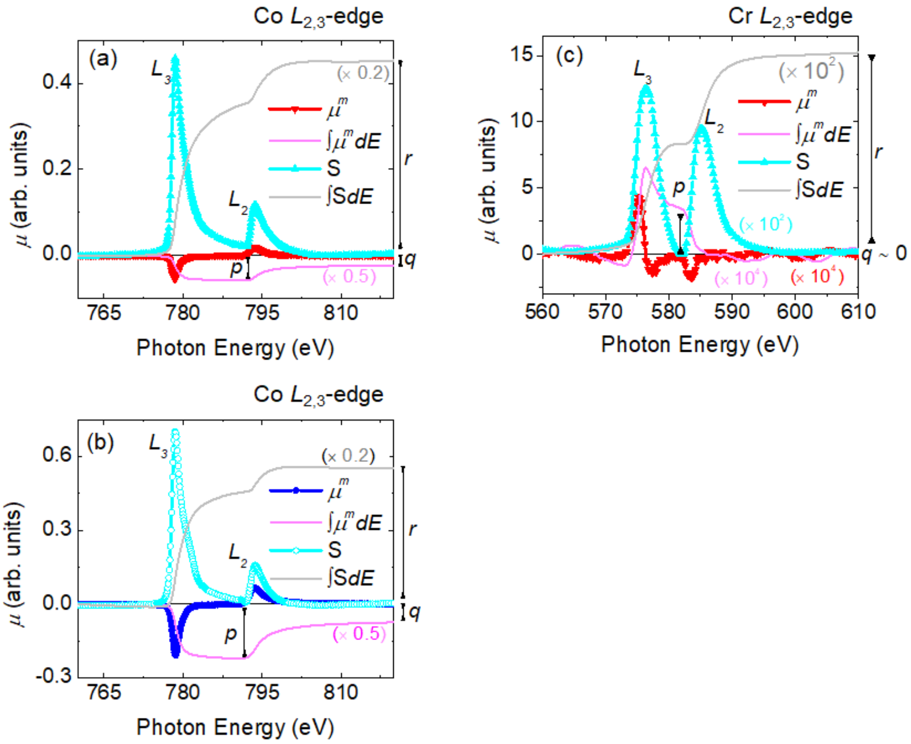

Fig. S8. Demonstration of sum rule for determination of spin and orbital angular momenta for the Co atom of (a) Cr_2N(5 nm)/Co(1 nm) and (b) Co(1 nm) films on the Al_2O_3 substrate (Fig. 5 in the main text). Similarly, for the Cr atom in the Cr_2N/Co film (c). Note that the sum of two XAS intensities (*S*) of opposite helicity was subtracted using a step function before integration.

§S9. Energy dependent spin/orbital Hall conductivity via first-principles calculation:

Spin-Hall conductivity ($\sigma_{ij}^{\mathrm{spin}(k)}$) and orbital-Hall conductivity ($\sigma_{ij}^{\mathrm{orb}(k)}$) of the Cr_2N supercell was calculated with the method addressed in the Experimental Section in the main text, where *i*, *j*, and *k* represent the direction of charge current, spin current, and polarization. We have found from Figs. 6(a1) and 6(a2) in the main text that the $\sigma_{xy}^{\mathrm{orb}(k)}$ was one or two orders of magnetude larger than $\sigma_{xy}^{\mathrm{spin}(k)}$ and the *z*-component of $\sigma_{xz}^{\mathrm{orb}(z)}$dominates the emtire orbital-Hall conductivity, while the spin in *y*-component of $\sigma_{xz}^{\mathrm{spin}(y)}$ dominates the entire spin-Hall conductivity for the conventional heavy-metals such as Pt, Ta, and W. This could be associated with the intrinsic properties and/or the crystalline inversion symmetry for the Cr_2N MXene, as also suggested for the recent TMDCs.[S9] To investigate the other possible components of $\sigma_{ij}^{\mathrm{spin}(k)}$and $\sigma_{ij}^{\mathrm{orb}(k)}$ in the Cr_2N, we calculated them for more three components. For (*i*, *j*) = (*y*, *z*), the *k* with *x*, *y*, and *z* components were evident for orbital-Hall conductivity [Figs. S9(b)]. The case with *i* = *y* (charge current orthogonal to the mirror plane) generally allows for the *k* = *z* due to the symmetry breaking of crystal structure.[S10] Therefore, the results in Figs. S9(b1) and S9(b2) can be understood by such exsisting mechanism, while cannot for the results on the (*i*, *j*) = (*x*, *z*) shown in Figs. 6(a1) and 6(a2) in the main text. These results led us to consider that the spin/orbital current with *z* polarization emerges in the Cr_2N MXene, regardless of the in-plane charge current directions either *x* or *y* with respect to the mirror axis, which would be the unique characteristics for the Cr_2N MXene. Figures S9(c) and S9(d) show the same results but for (*i*, *j*) = (*x*, *y*) and (*i*, *j*) = (*y*, *x*), respectively.We can see from these figures that $\sigma_{xy/yx}^{\mathrm{orb}(k)}$ was much larger than $\sigma_{xy/yx}^{\mathrm{spin}(k)}$and the orbital current with *k* = *z* is smaller than that with with *k* = *x* and *y*.

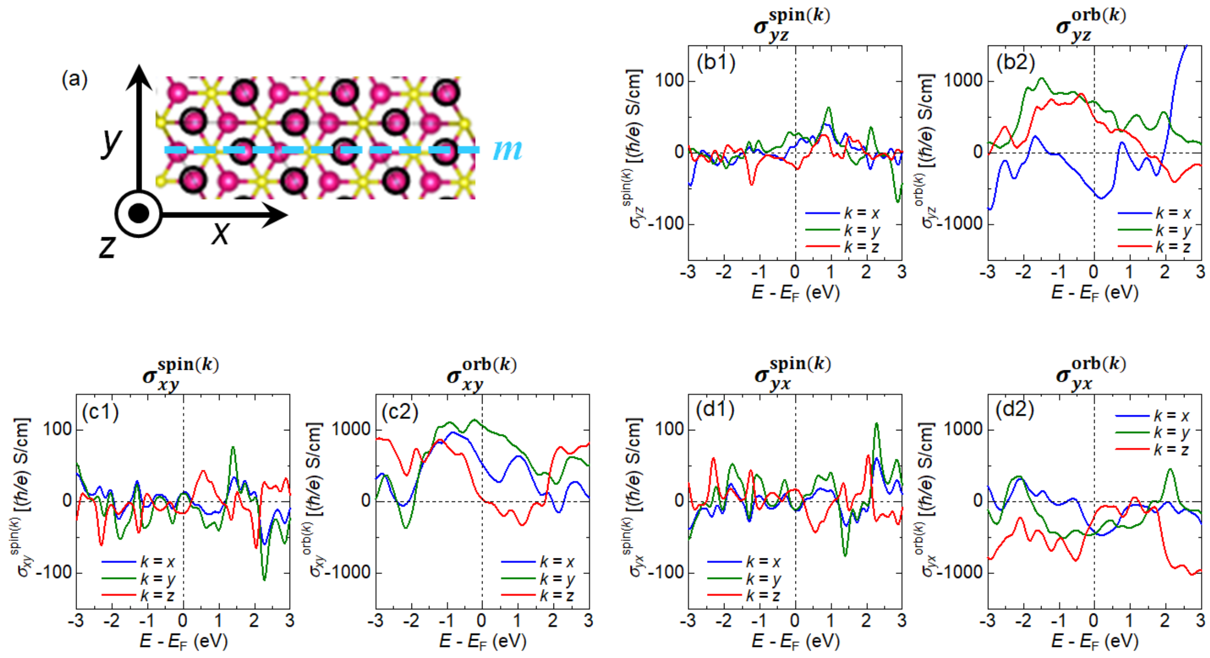

Fig. S9. (a) Definition of crystals and coordinates in first-principles calculations. (b-d) Energy dependent spin-Hall conductivity ($\sigma_{ij}^{\text{spin}(k)}$) and orbital-Hall conductivity ($\sigma_{ij}^{\text{orb}(k)}$) of the Cr_2N for (i, j) = (y, z) (b), (i, j) = (x, y) (c), and (i, j) = (y, x), where i, j, and k represent the direction of charge current, spin current, and polarization. The 3 × 3 × 1 supercell as depicted in Fig. 2(a3) was employed for the calculation.

§S10. Spin/orbital Hall conductivity-projected band structures and Berry curvatures via first-principles calculation:

Figures S10(a1)-(a5) show the band dispersion of Cr_2N along the high symmetry line, where the projected density of states (PDOS) for each *d* orbital of Cr atom in Cr_2N are highlighted by in each color. Furthermore, Figs. S10(b1) and (b2) show the spin and orbital Berry curvatures of Cr_2N along the high-symmetry line. The *Γ-K* line has relatively large spin and orbital Berry curvatures, which significantly contribute to the spin and orbital Hall conductivities of Cr_2N. We found that the band composed by *d*(*xy*) and *d*(*yz*) orbitals are crossing the Fermi level along the *Γ-K* line, which are mainly contributing to the spin and orbital Berry curvatures of Cr_2N.

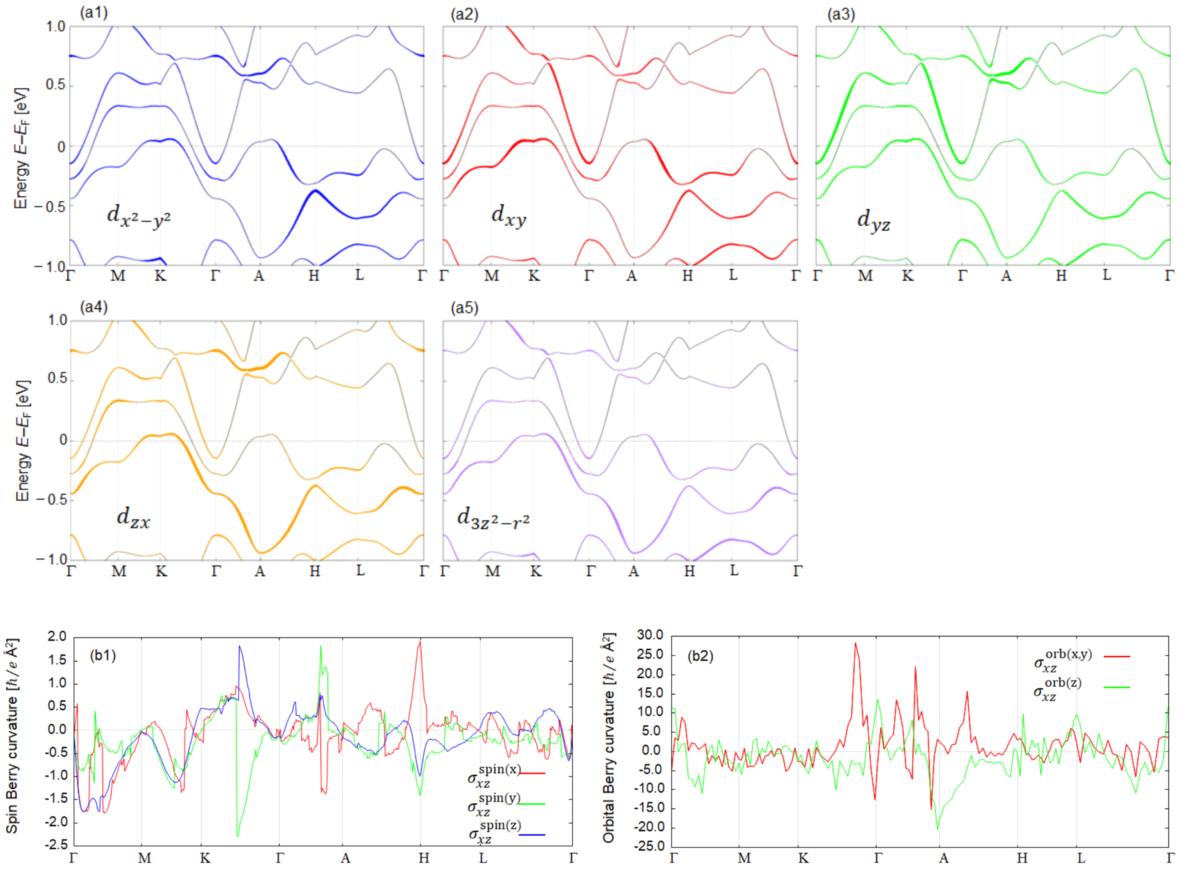

Fig. S10. (a1-a5) Band dispersion of Cr_2N along the high symmetry line for each *d* orbital. (b1,b2) Spin/orbital Berry curvatures of Cr_2N along the high symmetry line, where $\sigma_{ij}^{\mathrm{spin(k)/orb}(k)}$ denotes the spin/orbital-Hall conductivity with the *i* (*j*)-direction of charge (spin/orbital) current.

§S11. CIMS for SOT-device with 1-nm-Cu insertion, Cr_2N/Cu/[Co/Pt]$_3$:

To demonstrate the existence of a spin-filtering-like mechanism at the Cr_2N/(Co/Pt)$_3$ interface induced by the magnetization of Cr, the 1-nm-thick Cu layer was inserted between Cr_2N and (Co/Pt)$_3$ layers, namely, Al_2O_3 sub//Cr_2N(5 nm)/Cu(1 nm)/[Co(0.35 nm)/Pt(0.3 nm)]$_3$/MgO(3 nm). Due to its weak spin-orbit coupling and non-magnetism, Cu would serve as an ideal spacer material to isolate and identify the interfacial contribution to the observed field-free CIMS. Experimental study was conducted for both configurations, where the charge current direction is parallel [Fig. S11(a1)] (orthogonal [Fig. S11(b1)]) to the mirror symmetry axis. The out-of-plane and in-plane AHE for parallel configuration were consistent with those for orthogonal configuration as shown in Figs. S11(a2,a3,b2,b3), suggesting the same magnetic properties of (Co/Pt)$_3$ layer. Note that field dependent CIMS was observed for both configurations, while no field-free CIMS was evident by the Cu insertion [Fig. S11(a4,b4)]. The result is attributed to the absence of induced Cr moment. We thus conclude that magnetic moment of Cr induced by the Co at the interface plays an essential role for field-free CIMS, which may become a key to elucidate one of the possible scenarios of the spin-filtering mechanism with Cr moment.

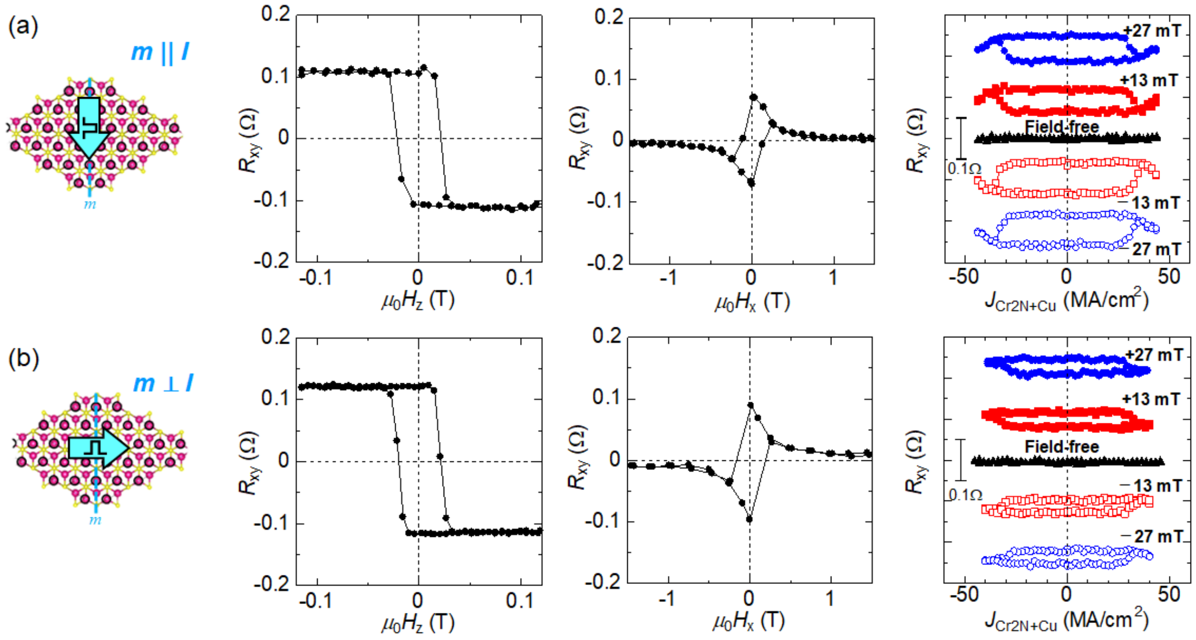

Fig. S11. (a,b) Out-of-plane and in-plane AHE, and CIMS results with two different measurement configurations, where current pulses are parallel (a) and orthogonal (b) to the mirror-symmetry line (*m*). The measurement sample has 1-nm-thick Cu layer and Hall-bar structure: substrate//Cr_2N(5 nm)/Cu(1 nm)/[Co(0.35 nm)/Pt(0.3 nm)]$_3$/MgO(3 nm).

References

[S1] S. Hashimoto, Y. Ochiai, and K. Aso. Perpendicular magnetic anisotropy and magnetostriction of sputtered Co/Pd and Co/Pt multilayered films. *J. Appl. Phys.* **1989**, 66, 4909.

[S2] M. A. Gharavi, G. Greczynski, F. Eriksson, J. Lu, B. Balke, D. Fournier, A. le Febvrier, C. Pallier, and P. Eklund. Synthesis and characterization of single-phase epitaxial Cr_2N thin films by reactive magnetron sputtering. *J. Mater. Sci.* **2019**, 54, 1434.

[S3] O. J. Lee, L. Q. Liu, C. F. Pai, Y. Li, H. W. Tseng, P. G. Gowtham, J. P. Park, D. C. Ralph, and R. A. Buhrman. Central role of domain wall depinning for perpendicular magnetization switching driven by spin torque from the spin Hall effect. *Phys. Rev. B* **2014**, 89, 024418.

[S4] X. Qiu, W. Legrand, P. He, Y. Wu, J. Yu, R. Ramaswamy, A. Manchon, and H. Yang. Enhanced Spin-Orbit Torque via Modulation of Spin Current Absorption. *Phys. Rev. Lett.* **2016**, 117, 217206.

[S5] D. Macneill, G. M. Stiehl, M. H. D. Guimaraes, R. A. Buhrman, J. Park, D. C. Ralph. Control of spin–orbit torques through crystal symmetry in WTe_2/ferromagnet bilayers. *Nat. Phys*. **2017**, 13, 300.

[S6] C. L. Prajapat, Surendra Singh, D. Bhattacharya, G. Ravikumar, S. Basu, S. Mattauch, J.-G. Zheng, T. Aoki, and A. Paul. Proximity effects across oxide-interfaces of superconductor-insulator-ferromagnet hybrid heterostructure. *Sci. Rep.* **2018**, 8, 3732.

[S7] J. Okabayashi, Y. Miura, and H. Munekata. Anatomy of interfacial spin-orbit coupling in Co/Pd multilayers using X-ray magnetic circular dichroism and first-principles calculations. *Sci. Rep.* **2018**, 8, 8303.

[S8] M. Magnuson, and M. Mattesini. Magnetic anisotropy in Cr_2GeC investigated by X-ray magnetic circular dichroism and ab initio calculations. *J. Magn. Magn. Mater.* **2020**, 501, 166470.

[S9] S. Bhowal, and S. Satpathy. Intrinsic orbital moment and prediction of a large orbital Hall effect in two-dimensional transition metal dichalcogenides. *Phys. Rev. B* **2020**, 101, 121112(R).

[S10] T. Nan, C. X. Quintela, J. Irwin, G. Gurung, D. F. Shao, J. Gibbons, N. Campbell, K. Song, S.-Y. Choi, L. Guo, R. D. Johnson, P. Manuel, R. V. Chopdekar, I. Hallsteinsen, T. Tybell, P. J. Ryan, J.-W. Kim, Y. Choi, P. G. Radaelli, D. C. Ralph, E. Y. Tsymbal, M. S. Rzchowski, and C. B. Eom. Controlling spin current polarization through non-collinear antiferromagnetism. Nature Communications **2020**, 11, 4671.
